# Circadian gene expression in adolescents: Associations with concurrent circadian disruption and subsequent changes in cardiometabolic risk measures

**DOI:** 10.1016/j.sleep.2026.108819

**Published:** 2026-02-05

**Authors:** Donghan Su, Jaclyn M. Goodrich, Jennifer T. Lee, Dana C. Dolinoy, Karen E. Peterson, Ronald D. Chervin, Helen J. Burgess, Martha María Téllez-Rojo, Alejandra Cantoral, Libni Torres-Olascoaga, Maricruz Tolentino, Peter X.K. Song, Louise M. O'Brien, Erica C. Jansen

**Affiliations:** a Department of Nutritional Sciences, University of Michigan School of Public Health, Ann Arbor, MI, USA; b Department of Environmental Health Sciences, University of Michigan School of Public Health, Ann Arbor, MI, USA; c Sleep Disorders Center and Department of Neurology, University of Michigan, Ann Arbor, MI, USA; d Department of Psychiatry, University of Michigan, Ann Arbor, MI, USA; e Center for Research on Nutrition and Health, National Institute of Public Health, Cuernavaca, Mexico; f Department of Health, Iberoamerican University, Mexico City, Mexico; g Department of Nutrition, National Institute of Perinatology, Mexico City, Mexico; h Department of Biostatistics, University of Michigan School of Public Health, Ann Arbor, MI, USA

**Keywords:** Circadian disruption, Sleep timing, Social jetlag, Clock gene expression, Cardiometabolic health, Adolescents

## Abstract

**Objectives:**

Circadian disruption has been linked to adverse metabolic health. Adolescents are particularly susceptible to circadian disruptors, such as delayed sleep onset and social jetlag, which may have sex-specific effects. However, evidence linking these disruptors with circadian gene expression and subsequent cardiometabolic risk remains limited.

**Methods:**

Our study included 203 adolescents (53% females, median age 13.6 years) from the ELEMENT cohort in Mexico City. Sleep was assessed via 7-day wrist actigraphy. A fasting venipuncture blood sample was collected between 8:00 a.m. and 12:00 p.m. RNA was isolated from blood leukocytes and sequenced to determine the relative expression of genes. We conducted differential gene expression analysis for 12 core clock genes in relation to sleep midpoint and social jetlag, adjusting for sleep duration and other potential confounders. We further evaluated how circadian gene expression associated with changes in adiposity, glucose metabolism, blood pressure, and lipid profiles over two years using linear regression.

**Results:**

Later sleep midpoint (per 1-h increase) was associated with reduced mid-morning expression of four circadian genes: *RORA* (log2 fold change [LFC]: −0.190; *P* value: 0.001), *RORC* (LFC: −0.147; *P* value: 0.039), *CLOCK* (LFC: −0.141; *P* value: 0.019), and *NR1D2* (LFC: −0.093; *P* value: 0.029). Additionally, expression levels of several clock genes (*CRY1, NR1D2, BMAL1,* and *PER1-3*) were associated with changes in metabolic biomarkers over two years in sex-specific patterns. For instance, *NR1D2* showed a negative association with fasting glucose among females (β: −0.0012; *P* value: 0.020), while demonstrating positive associations with LDL cholesterol (β: 0.0023; *P* value: 0.002) and total cholesterol (β: 0.0016; *P* value: 0.028) among males.

**Conclusions:**

Expression of core clock genes was linked to circadian disruption and changes in cardiometabolic risk factors in a sex-specific manner among adolescents. Our findings provide novel insights into potential biological mechanisms underlying associations of circadian disruption with cardiometabolic health.

## 1. Introduction

The global rise in cardiometabolic disorders among youth represents a significant public health challenge. Recent estimates suggest that approximately 125 million children and adolescents aged 5–19 years are affected by obesity, with a concomitant rise in insulin resistance and hypertension among youth [1-3]. Adverse cardiometabolic conditions that develop or worsen during adolescence have implications for elevated long-term risk of metabolic syndrome and cardiovascular diseases in adulthood, highlighting the need for a better understanding of the factors driving these trends [4].

Circadian rhythms are timed daily cycles that evolved to synchronize biological processes with the external environment. Circadian disruption—defined as the misalignment between intrinsic biological rhythms and external environmental or behavioral cues such as sleep timing, feeding patterns, and artificial lighting—has been increasingly linked to adverse metabolic outcomes [5,6]. Adolescents are particularly vulnerable to circadian disruptions due to physiological changes co-occurring with puberty that favor later sleep patterns, including late sleep onset and midpoint (median of bedtime and wake time). Importantly, sleep timing on weekdays is often constrained by early school schedules and may therefore reflect accumulating circadian misalignment between endogenous circadian phase and social demands. In addition, differences in weekday vs weekend schedules and activities often lead to greater social jetlag, defined as differences in sleep midpoint between weekdays and weekends [7]. Prior studies have indicated that sleep timing and social jetlag may play critical roles in adolescent metabolic health independently of sleep duration. In particular, our group previously showed that Mexican adolescents with later sleep midpoint had higher prevalence of insulin resistance as well as greater odds of becoming insulin resistant over a two-year period, even after accounting for sleep duration [8]. Moreover, we reported distinct DNA methylation signatures for sleep midpoint as compared to sleep duration [9]. However, the underlying biological mechanisms governing relationships between circadian disruption and metabolic health during adolescence remain unclear [7,10,11].

At the molecular level, circadian rhythms are governed by an intricate network of core clock genes, such as *CLOCK, BMAL1, PER*, and *CRY*, which orchestrate the rhythmic expression of thousands of genes involved in metabolic regulation throughout the body [12,13]. Animal models have shown that genetic manipulation of these clock genes can lead to impaired glucose tolerance, insulin resistance, hyperlipidemia, hypertension, and obesity, highlighting their potential causal role in metabolic dysfunction [14,15]. Although human studies are limited, emerging evidence suggests that misalignment of circadian rhythm may disrupt the expression of these clock genes and leads to metabolic dysfunctions, shedding light on the biological pathways through which circadian disruption may contribute to metabolic health. For example, previous studies have observed decreased morning expression and reduced amplitudes of nearly all core clock genes among rotating shift nurses compared to day shift nurses [16,17]. Additionally, intervention studies in healthy adults demonstrated short-term circadian misalignment could decrease the mean amplitude of circadian gene transcripts, down-regulate the expression levels of *CLOCK* and *RORA* [18], and result in elevated blood glucose and free fatty acid levels [19]. However, such studies are limited in number, often cross-sectional, and conducted among adult populations. The extent to which sleep timing and social jetlag influence circadian gene expression in adolescents, and whether these relationships differ by sex, remains poorly understood. Our prior work in the ELEMENT cohort showed sex-specific associations between sleep duration and timing with insulin resistance, as well as distinct DNA methylation signatures of sleep among boys and girls, underscoring the need to explore molecular mechanisms underlying these sex differences [9,20]. Moreover, existing research relies predominantly on small-size intervention studies to examine gene expression levels and metabolic outcomes in the short term, limiting their ability to understand potential longer-term metabolic consequences.

This study aims to address these gaps by investigating the relationship between objectively measured sleep timing and social jetlag, circadian gene expression, and cardiometabolic risk factors among Mexican adolescents from the ELEMENT cohort. Specifically, we examined the cross-sectional associations of sleep timing and social jetlag with expression of 12 core clock genes, including *BMAL1, CLOCK, PER1, PER2, PER3, CRY1, CRY2, NR1D1, NR1D2, RORA, RORB*, and *RORC*. We then assessed how these circadian genes were prospectively linked to cardiometabolic risk factors measured approximately two years later. By leveraging objectively measured sleep parameters and robust RNA-sequencing methods in a well-characterized cohort, this study aimed to uncover potential molecular mechanisms underlying associations between circadian disruption and adolescent cardiometabolic health.

## 2. Methods

### 2.1. Study population

This study was conducted within the Early Life Exposure in Mexico to Environmental Toxicants (ELEMENT) cohort, a prospective birth cohort study based in Mexico City. The ELEMENT study design and participant recruitment have been described in detail previously [21]. Briefly, between 1997 and 2004, 1012 mother/child dyads were recruited from maternity hospitals serving low-to middle-income populations during the first trimester of pregnancy. Their children were followed periodically from birth through adolescence. A subset of 554 adolescents aged 9 to 17 years who were amid pubertal transition participated in a follow-up study in 2015 (T1). Then in 2017, the same participants were recruited for a second follow-up study (T2, n = 519). Participants received modest compensation for each study visit (i.e. grocery vouchers or gifts worth $20–30), and transportation support was provided to all participants when needed (i.e., arranged car pick-up). During the study visit, researchers collected questionnaires on sociodemographic, lifestyle, and health information, took anthropometric assessments, collected fasting blood samples, and provided instructions on the 7-day wrist actigraphy.

For the present study, we included 203 adolescents who had RNA sequencing data that passed quality control at T1 visit. Sample sizes slightly varied when using changes in different cardiometabolic risk factors over the two-year period as outcomes (Supplemental Fig. 1). All study procedures were approved by the Institutional Review Boards of the National Institute of Public Health in Mexico and the University of Michigan. Written informed consent was obtained from all participants, and parental consent was required for minors.

### 2.2. Sleep timing and social jetlag assessment

Sleep parameters were assessed using wrist-worn ActiGraph GT3X-BT accelerometers (ActiGraph LLC, Pensacola, FL). Participants were instructed to wear the device continuously on their non-dominant wrist for seven consecutive days, removing it only for water-based activities. Nighttime sleep measurements were estimated from the actigraphy data with 60-s epoch lengths using a pruned dynamic programming (PDP) algorithm in R [22]. This algorithm incorporated self-reported bedtimes and wake times to refine the sleep-wake detection and has been previously validated against polysomnography.

The primary sleep exposure was average weekday sleep midpoint at T1, calculated as the median time between sleep onset and wake time on weekdays (reported in decimal hours). Social jetlag was defined as the absolute difference between average sleep midpoint on weekdays and weekends. Average weekday sleep duration at T1 was also recorded and included as an adjustment variable in regression models.

### 2.3. Gene expression assessment

For the present analysis, the study focused on twelve core circadian genes, including *BMAL1, CLOCK, CRY1, CRY2, NR1D1, NR1D2, PER1, PER2, PER3, RORA, RORB,* and *RORC*. Mid-morning whole blood samples were collected at the T1 study visit via venipuncture after an overnight fast of at least 8 h. White blood cells were spun down and stored in RNAlater at −80 °C until RNA isolation in 2023 at the University of Michigan. The RNA was isolated from leukocytes using the Maxwell RSC simplyRNA tissue kit (Maxwell; catalog # AS1340) and underwent quality assessment using an Agilent Bioanalyzer. Samples (n = 216) with an RNA Integrity Number (RIN) ≥ 7 were retained for sequencing [23]. Library preparation was performed using the Universal Plus mRNA-Seq with NuQuant, Human globin AnyDeplete (NuGEN Technologies, Inc.; now sold by Tecan) which depletes globin transcripts prior to sequencing. Quality of the library preparations was checked, and 207 high quality samples were selected for sequencing. Sequencing was conducted on the Illumina NovaSeqXPlus platform, generating 151 bp paired-end stranded mRNA sequences with a median sequencing depth of 39.7 million reads per sample [23]. Raw sequencing data were processed using BCL Convert Conversion Software v4.0 (Illumina) to generate demultiplexed Fastq files. Quality control and alignment were performed using FastQC, with additional filtering conducted to identify and remove samples with unexpected Y chromosome gene expression patterns, yielding a final analytic sample of 203 participants. All samples were processed at the same core facility as part of a single coordinated library preparation and sequencing effort using identical protocols, with randomized sample processing order and flow-cell assignment; thus, technical batch effects are expected to be minimal. Because only samples with RNA Integrity Number (RIN) ≥ 7 were included (range 7–10), the impact of RNA quality on expression of the core circadian genes examined is expected to be negligible.

Differential gene expression analyses were conducted using DESeq2 [24], which normalizes counts for library sizes and read depth via the default median-of-ratios size-factor method under a negative binomial model. For downstream regression analyses of gene expression and cardiometabolic outcomes, normalization of gene expression was conducted using the variance-stabilizing transformation (VST) method to account for differences in library sizes, read depth, and gene-specific dispersion. To ensure robust analysis, genes with a median expression count of <10 across all samples were excluded (*RORB*). The final dataset contained normalized expression values for 11 core clock genes (*BMAL1, CLOCK, CRY1, CRY2, NR1D1, NR1D2, PER1, PER2, PER3, RORA,* and *RORC*), which were analyzed in association with sleep parameters and metabolic biomarkers.

### 2.4. Cardiometabolic risk factors

We assessed 11 cardiometabolic risk factors at two study visits (T1 and T2) and used their changes from T1 to T2 (T2-T1) in our analyses. These change scores were subsequently standardized to mean 0 and standard deviation 1 for analysis. Details on biomarkers assessment in this cohort can be found in a previous publication [25]. Anthropometric measures included body mass index (BMI) and waist circumference (WC). BMI was calculated as weight measured to the nearest 0.1 kg divided by square of height measured to the nearest 0.1 cm (converted into m^2^ for calculation), and WC was measured to the nearest 0.1 cm. Both were measured two times (or three times if the first two measurements have large discrepancy) following standardized protocols and the average of these measurements was used in our study.

Systolic blood pressure (SBP) and diastolic blood pressure (DBP) were measured using an automated oscillometric monitor following standardized procedures. Two seated measurements were taken at 1-min intervals, and the average of the two readings was used for our analysis. Fasting blood samples were collected after an overnight fast and analyzed for metabolic biomarkers. Fasting plasma glucose concentration was determined using the final point colorimetric enzymatic method with an automated biochemistry analyzer (Respons 910, Diasys Halzeim, Germany). Fasting insulin levels were measured using via enzyme-linked immunosorbent assay chemiluminescence method (Immulite ^®^ 1000 S Llanberis Gwynedd, United Kingdom) [26]. Insulin resistance was estimated using the Homeostatic Model Assessment of Insulin Resistance (HOMA-IR), calculated as fasting insulin (μU/L) × fasting plasma glucose (mmol/L)/22.5 [27]. Lipid profile assessment included total cholesterol, high-density lipoprotein cholesterol (HDL-C), low-density lipoprotein cholesterol (LDL-C), and triglycerides. Total cholesterol, HDL-C, and triglycerides were quantified by a biochemical analyzer using the final point colorimetric enzymatic method with an automated biochemistry analyzer (Respons 910, Diasys Halzeim, Germany).

### 2.5. Covariates

Potential confounders were selected a priori, including age, sex, pubertal status, education level of the head of the household, socioeconomic status, smoking status, drinking status, sedentary time, moderate-to-vigorous physical activity (MVPA), weekday sleep duration, and InBody assessment time. Pubertal status was determined using orchidometer measurements of testicular volume for males following standard protocols and menarche status for females, obtained through an interviewer-administered questionnaire. Pubertal status at the T1 visit was dichotomized into earlier stages (testicular volume <15 mL for males or being premenarcheal for females) and later/post-pubertal stages (testicular volume ≥15 mL for males or having reached menarche for females) [28]. Sociodemographic and lifestyle characteristics were self-reported through questionnaires. Education level and socioeconomic status were reported by mothers. Education level of the head of the household was categorized into three levels: less than high school, some high school or undergraduate, and undergraduate degree or above. Household socioeconomic status was estimated based on housing conditions, number of rooms, type of toilet, electricity access, and asset ownership, and was categorized into six levels [29]. Smoking status was dichotomized as never smoked or ever smoked. Lifetime alcohol use was assessed via a self-reported question asking whether participants had ever consumed an alcoholic beverage and was dichotomized as yes/no. Sedentary time, moderate-to-vigorous physical activity, and weekday sleep duration were estimated using actigraphy. Sedentary time and moderate-to-vigorous physical activity were categorized according to the validated Chandler's Vector Magnitude cutoffs in minutes per day [30], while weekday sleep duration (in hours) was averaged across weekdays. Researchers recorded the time of the InBody (bioelectrical impedance) assessment, which approximated when the blood samples were drawn.

### 2.6. Statistical analysis

Among these 203 participants, 9 individuals had missing measurements for covariates (i.e. smoking status, drinking status, sedentary time, MVPA, or time of the InBody assessment as a proxy for clock time of blood draw) measured at T1. To handle missing data, we applied multiple imputation using chained equations with 10 imputations. Because DESeq2 does not directly support pooling of estimates across multiply imputed datasets, a single completed dataset was constructed by summarizing continuous variables using the median and categorical variables using the mode across imputed datasets. We summarized the baseline characteristics of participants according to sleep midpoint (<4 a.m. vs. ≥ 4 a.m.) and social jetlag (<2 h vs. ≥ 2 h), using cutoffs from previous studies [8,31,32]. The mean or median was used to summarize continuous variables, while percentages were used for categorical variables. Differences by sleep parameters were assessed using chi-square tests for categorical variables and independent t-tests for continuous variables. Spearman's rank correlation analysis was performed to assess inter-gene correlations among 11 core clock genes (*BMAL1, CLOCK, CRY1, CRY2, NR1D1, NR1D2, PER1, PER2, PER3, RORA,* and *RORC*).

We examined the association of sleep midpoint and social jetlag, modeled as continuous variables, with mid-morning circadian gene expression using DESeq2 [24], which employs a generalized linear model based on the negative binomial distribution, adjusting for age, sex, pubertal status, education level of the head of the household, socioeconomic status, smoking status, drinking status, sedentary time, moderate-to-vigorous physical activity, weekday sleep duration, and InBody assessment time. Main analyses were repeated separately in females and males to assess potential effect modification by sex. As a post-hoc analysis, we further adjusted for the month of the study visit at T1, in addition to the covariates in the main model, to account for potential seasonal effects on circadian gene expression. To evaluate potential confounding by leukocyte subset composition, we first tested Spearman rank correlations between VST-transformed expression levels of each core clock gene and proportions of neutrophils, lymphocytes, monocytes, eosinophils, and basophils. We found that neutrophil, lymphocyte, and eosinophil proportions show modest correlations with several core clock gene expression (∣ρ∣ generally ≤0.3). Because neutrophil and lymphocyte proportions were strongly inversely correlated, neutrophils were selected as the primary marker of major leukocyte composition to minimize collinearity, and eosinophils were additionally included based on observed correlations. Sensitivity analyses were conducted by repeating the main DESeq2 models with additional adjustment for neutrophil and eosinophil proportions.

Next, we assessed the association between normalized circadian gene expression (exposure) and standardized changes in cardiometabolic risk factors from T1 to T2 (outcome: BMI, WC, fasting glucose, fasting insulin, HOMA-IR, SBP, DBP, total cholesterol, HDL-C, LDL-C, and triglycerides) using linear regression models. These models were adjusted as described above, with additional adjustment for height in models where systolic or diastolic blood pressure was the outcome. We further adjusted for the month of the study visit at T1 in addition to the covariates in the main model as a post-hoc analysis. We also further adjusted for adherence to three dietary patterns, identified at T1 in a previous study using principal component analysis. [33]. Analyses were then repeated separately in females and males to evaluate potential effect modification by sex.

To explore potential non-linear associations between circadian gene expression and metabolic biomarkers, restricted cubic spline models with four knots were applied. Significant deviation from linearity was assessed using likelihood ratio tests, comparing models with and without the restricted cubic spline term.

Due to the exploratory nature of our analyses, a *P* value < 0.05 was considered statistically significant (i.e., not adjusted for multiple comparisons). All analyses were performed in R version 4.4.1 (R Core Team 2024) and Stata version 17.0 (StataCorp, LLC).

## 3. Results

### 3.1. Participant characteristics

The study included 203 participants with a median age of 13.6 years (interquartile range: 12.1–15.6) (Table 1a). Participants with a later sleep midpoint (≥4:00 a.m.) were significantly older (14.3 vs. 13.2 years, *P* value < 0.001) and more likely to have reached puberty (*P* value: 0.003). They also exhibited longer weekday sleep duration (9.1 vs. 8.1 h, *P* value < 0.001) and engaged in less moderate-to-vigorous physical activity (*P* value: 0.017). Participants with a later sleep midpoint were more likely to have ever smoked (*P* value: 0.002), but there were no significant differences in alcohol or substance use between groups. The distribution of social jetlag (absolute values) was narrow, with most participants exhibiting relatively small-to-moderate weekday–weekend differences (Supplemental Fig. 2–1). Examination of signed social jetlag values (weekend minus weekday sleep midpoint) showed that most participants had later sleep timing on weekends, consistent with the expected adolescent pattern (Supplemental Fig. 2–2). Social jetlag ≥2 h was associated with significantly shorter weekday sleep duration (*P* value < 0.001), but no significant differences in age, sex, or socioeconomic indicators were observed (Table 1b). The InBody assessment time was between 8:02 and 11:42 with average study visit at 9:21 (Supplemental Fig. 3) (Table 1a).

### 3.2. Sleep timing, social jetlag, and circadian gene expression

Spearman's rank correlation analysis revealed positive correlations among all core clock gene pairs, except between *PER1* and *RORC* (ρ = −0.19) (Supplemental Fig. 4). A later sleep midpoint (per 1-h increase) was associated with reduced mid-morning expression of four circadian genes after adjusting for age, sex, pubertal status, smoking status, drinking status, sedentary time, moderate-to-vigorous physical activity, education level of head of the household, socioeconomic status, weekday sleep duration, and InBody assessment time: *RORA* (β: −0.190; *P* value: 0.001), *RORC* (β: −0.147; *P* value: 0.039), *CLOCK* (β: −0.141; *P* value: 0.019), and *NR1D2* (β: −0.093; *P* value: 0.029) (Table 2). The β estimates remained largely unchanged after further adjusting for the month of study visit; however, the association with *NR1D2* was no longer statistically significant (Supplemental Table 1). The results were largely unchanged after additionally adjusting for neutrophil and eosinophil proportions, but *RORC* became marginally statistically significant (Supplemental Table 2). Unadjusted analyses showed negative associations between sleep midpoint and gene expression with two more genes, *CRY1* (β: −0.132; *P* value: 0.013) and *CRY2* (β: −0.059; *P* value: 0.033), but associations were attenuated and no longer significant after adjustment for confounders (Supplemental Table 3). In contrast, greater social jetlag (per 1-h increase) was associated with higher expression of circadian genes. For example, in the unadjusted model, social jetlag displayed a significant positive association with *RORC* expression (β: 0.199; *P* value: 0.033) (Supplemental Table 3). However, none of the associations remained statistically significant after adjusting for covariates (Table 2). Results were largely unchanged after adjusting for the month of the study visit (Supplemental Table 1) or adjusting for neutrophil and eosinophil proportions (Supplemental Table 2). When using signed social jetlag (weekend minus weekday sleep midpoint), associations with core clock gene expression were generally weaker than those observed using absolute social jetlag, except for *PER*1 (Supplemental Table 4).

In sex-stratified analysis, later sleep midpoint was significantly associated with lower *RORA* expression among girls only (β: −0.255; *P* value: 0.002) (Table 3). Additionally, greater social jetlag was associated with increased *PER1* expression in girls (β: 0.275; *P* value: 0.017), while no significant associations were observed in boys.

### 3.3. Circadian gene expression and metabolic biomarkers

**Fig. 1.**
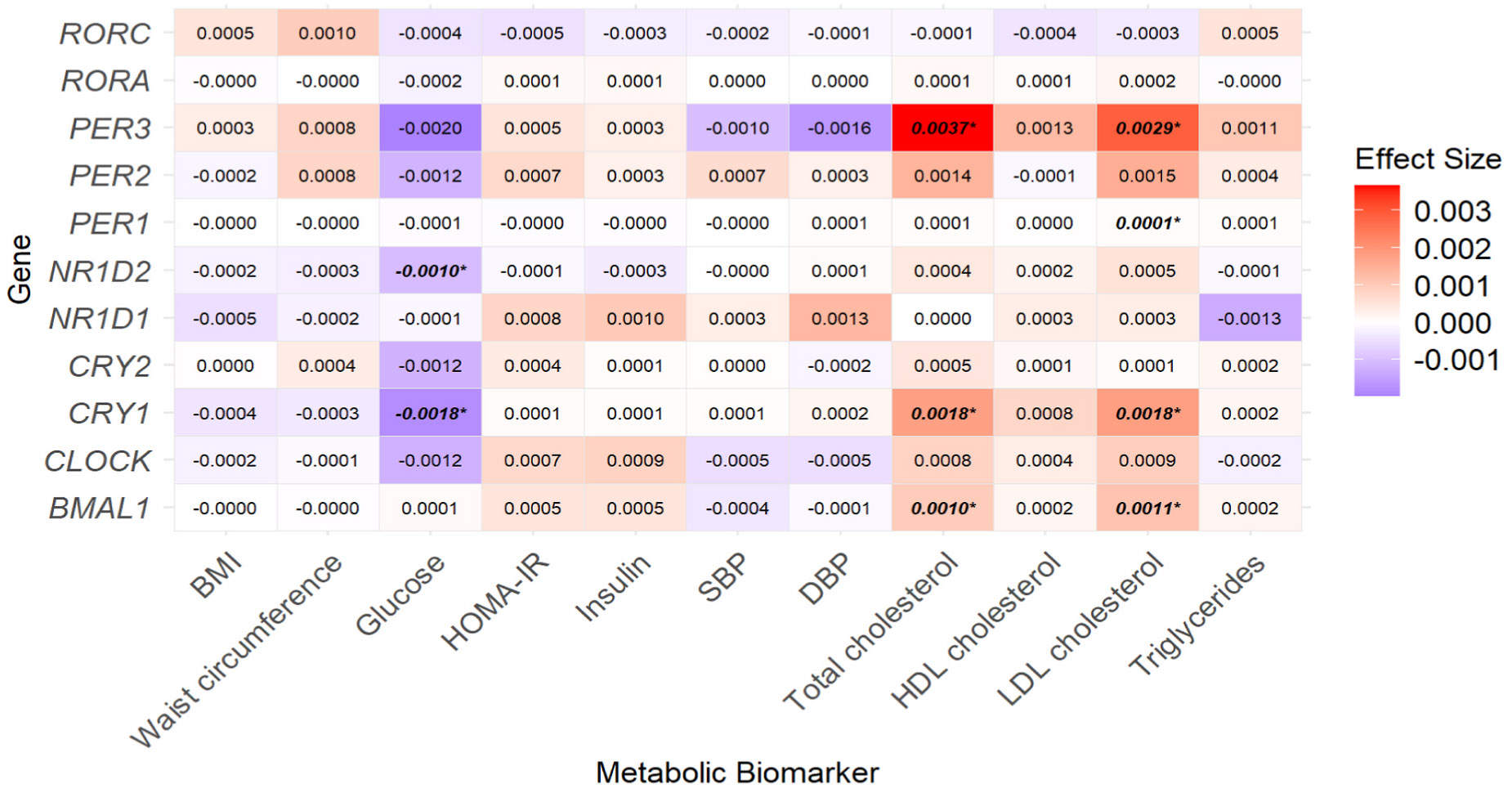
Associations between morning core clock gene expression and changes in metabolic biomarkers over a two-year period. Cardiometabolic risk factors were standardized to have a mean of 0 and standard deviation of 1. Models are adjusted for age, sex, puberty onset, smoking status, drinking status, sedentary time, moderate to vigorous physical activity time, education level of head of the household, socioeconomic status, weekday sleep duration, and InBody assessment time. Models were additionally adjusted for height when using systolic or diastolic blood pressure as the outcomes. Effect sizes are represented by colors and color gradients. Results shown with * are statistically significant at alpha level of 0.05.

The linear associations between circadian gene expression (exposure) and changes in cardiometabolic risk factors (outcome) from T1 to T2 are shown in Fig. 1 and Supplemental Table 5. Models were adjusted as indicated in the above analyses and were additionally adjusted for height when SBP or DBP was used as the outcome. Higher mid-morning expression of core clock genes at T1 exhibited negative associations with in fasting glucose levels changes two years later; however, only *CRY1* (β: −0.018; *P* value: 0.046) and *NR1D2* (β: −0.0010; *P* value: 0.017) reached statistical significance. In contrast, core clock gene expression tended to show positive associations with changes two years later in cholesterol level. Three genes demonstrated statistically significant associations with changes in total cholesterol and LDL cholesterol: *CRY1* (β: 0.0018 and *P* value: 0.039 for total cholesterol; β: 0.0018 and *P* value: 0.032 for LDL cholesterol), *BMAL1* (β: 0.0010 and *P* value: 0.048 for total cholesterol; β: 0.0011 and *P* value: 0.019 for LDL cholesterol), and *PER3* (β: 0.0037 and *P* value: 0.011 for total cholesterol; β: 0.0029 and *P* value: 0.043 for LDL cholesterol). Results were largely unchanged after additionally adjusting for dietary patterns at T1 (Supplemental Fig. 5) or the month of the study visit at T1 (Supplemental Fig. 6).

**Fig. 2.**
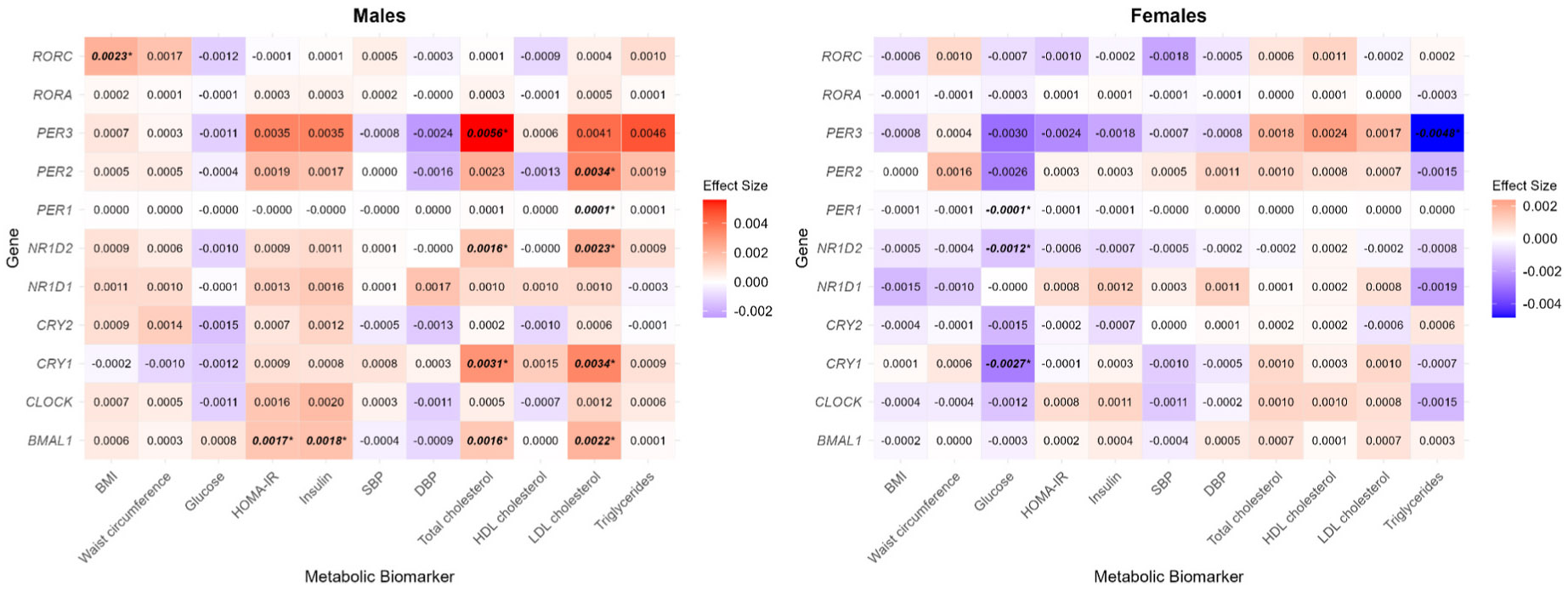
Associations between morning core clock gene expression and changes in metabolic biomarkers among the male (left) and females (right). Cardiometabolic risk factors were standardized to have a mean of 0 and standard deviation of 1. Models are adjusted for age, puberty onset, smoking status, drinking status, sedentary time, moderate to vigorous physical activity time, education level of head of the household, socioeconomic status, weekday sleep duration, and InBody assessment time. Models were additionally adjusted for height when using systolic or diastolic blood pressure as the outcomes. Effect sizes are represented by colors and color gradients. Results shown with * are statistically significant at alpha level of 0.05.

Sex-stratified analyses revealed distinct patterns. Among boys, *BMAL1* expression was significantly positively associated with changes in HOMA-IR (β: 0.0017; *P* value: 0.042) and fasting insulin level (β: 0.0018; *P* value: 0.028) over the two-year period (Fig. 2) (Supplemental Table 6). However, these associations were no longer statistically significant when additionally adjusting for dietary patterns at T1 (Supplemental Fig. 7). Additionally, the expressions of *BMAL1* (β: 0.0016; *P* value: 0.039), *CRY1* (β: 0.0031; *P* value: 0.026), *PER3* (β: 0.0056; *P* value: 0.010), and *NR1D2* (β: 0.0016; *P* value: 0.028) were significantly positively associated with changes in total cholesterol levels. Similarly, positive associations were found between *BMAL1, CRY1, NR1D2, PER1,* and *PER2* expression and changes in LDL cholesterol levels. In contrast, among girls, *CRY1* (β: −0.0027; *P* value: 0.021), *NR1D2* (β: −0.0012; *P* value: 0.020), and *PER1* (β: −0.0001; *P* value: 0.008) were significantly negatively associated with changes in fasting glucose. Additionally, a strong negative association was found between *PER3* expression levels and changes in triglycerides among the girls (β: −0.0048; *P* value: 0.027).

### 3.4. Non-linear associations between gene expression and metabolic biomarkers

Exploratory analyses examining potential non-linear relationships between circadian gene expression and metabolic biomarkers revealed significant deviations from linearity for several associations (Supplemental Fig. 8). Models were adjusted for covariates as indicated in the above analyses. A significant J-shaped association was observed between *PER1* expression at T1 and changes in both BMI (*P* value < 0.001) and waist circumference (*P* value: 0.036) two years later. Similarly, a J-shaped association was detected between *PER2* expression and changes in SBP, though the association was only marginally significant (*P* value: 0.053). Additionally, *PER3* expression demonstrated a significant non-linear association with changes in DBP (*P* value: 0.028).

## 4. Discussion

This study among adolescents in the ELEMENT cohort provides novel evidence linking adolescent sleep timing and social jetlag with concurrent circadian gene expression in blood leukocytes, and in turn, circadian gene expression with metabolic changes 2 years later. We observed that a later sleep midpoint was significantly associated with lower morning expression of several core clock genes, notably *RORA, RORC, CLOCK*, and *NR1D2*. Additionally, baseline expression levels of core clock genes (*CRY1, NR1D2, BMAL1*, and *PER1*–*3*) were associated with subsequent changes in metabolic biomarkers over two years. Notably, *NR1D2* and *CRY1* expression were linked to both sleep timing and longitudinal changes in fasting glucose, suggesting a potential molecular pathway between circadian disruption and cardiometabolic health. Finally, our findings revealed pronounced sex-specific patterns, as associations between sleep midpoint and social jetlag with circadian gene expression appeared to be stronger in girls. Moreover, there were sex-specific patterns in the associations between circadian gene expression and changes in cardiometabolic biomarkers, with significant negative associations observed between multiple circadian genes and fasting glucose and triglyceride levels in girls, and significant positive associations between several of the same genes and LDL cholesterol among boys, suggesting possible sex differences in susceptibility to circadian disruption and its metabolic consequences.

Our findings closely align with the growing evidence that circadian disruption in humans can adversely affect metabolic health. Adolescence is a life stage characterized by a physiologically driven delay in sleep timing, often resulting in social and academic schedules at odds with their circadian biology [7]. Prior studies have shown that this circadian disruption is linked to obesity and metabolic dysfunction in youth. For example, evening chronotypes and social jetlag correlate with greater adiposity in adolescent girls [7], and we previously showed within this same cohort of Mexican adolescents that the adolescents within the latest category of sleep midpoint were more likely to have insulin resistance compared to those with earlier sleep midpoints [20]. We now provide molecular evidence that adolescents’ sleep schedules are reflected in their circadian transcriptome, which in turn may be linked with metabolic changes two years later, particularly among females. This is, to our knowledge, one of the first studies to connect adolescent sleep timing with circadian gene expression and downstream metabolic profiles over time, offering new insight into how circadian disruption might translate into cardiometabolic risk during this critical developmental period.

Our observation that adolescents with later sleep timing have reduced *RORA, RORC, CLOCK*, and *NR1D2* expression is in line with previous literature on circadian misalignment. Disrupted or mistimed sleep can lead to circadian misalignment, which in adults has been shown to be associated with a dampening of the amplitude of clock gene transcription and acute metabolic disturbances. Previous studies among rotating shift nurses reported decreased morning expression of nearly all core clock genes compared to day shift nurses [16]. Intervention studies among adults demonstrated short-term circadian misalignment could decrease the mean amplitude of circadian gene transcripts, and down-regulate the expression levels of *CLOCK* and *RORA* [18]. Our observation suggests a similar mechanism: the suppression of clock gene expression associated with later sleep midpoints may reflect insufficient alignment between sleep schedules and the internal clock. For example, *RORA* is a transcriptional activator of *BMAL1* and its expression level normally rises during day [34]. A delayed sleep schedule likely reflects a delayed circadian phase, such that at a fixed morning clock time, the oscillation of *RORA* (and other genes like *CLOCK* or *NR1D2*) is lagged and may have a decreased amplitude. Prior work has shown that extreme evening-type individuals had a ~3-h delay in the peak of *PER3* and *NR1D2* expression compared to morning types [35]. Thus, the lower morning expression of these genes in late-sleeping adolescents may indicate an overall phase-delayed circadian clock or lower amplitude of rhythmic gene expression. Future work that includes repeated measures of gene expression across the day are needed to explore this hypothesis.

However, it was notable that, in contrast to sleep midpoint, social jetlag exhibited a positive relationship (though statistically non-significant in fully adjusted models) with core clock gene expression levels. A similar unexpected pattern was previously reported among Hispanic/Latino youth in the US, where higher social jetlag was associated with more favorable health behaviors and a lower likelihood of being overweight, whereas shorter sleep duration correlated with less favorable health outcomes [36]. This suggests that social jetlag and delayed sleep midpoint are distinct constructs and may differentially impact the internal circadian clock and subsequent metabolic health. Interpretation of social jetlag findings should be cautious given the restricted variability of differences in sleep midpoint in the weekend compared to weekdays in this cohort, which may have attenuated associations. Moreover, social jetlag was calculated as an absolute difference in this study, and positive (later weekend sleep midpoint relative to weekdays) and negative social jetlag may have differential influences on circadian gene expression. Future research should further clarify how irregular sleep schedule, captured by social jetlag, versus consistently delayed sleep timing influence circadian gene dynamics and metabolic health outcomes.

We also found evidence that expression of circadian genes was related to changes in several cardiometabolic biomarkers over a two-year period. RORs are also key regulators of metabolic gene networks. RORα and RORγ control the circadian expression of genes involved in lipid and glucose metabolism [37]. A disruption of human *RORA_1_* was found to be present in families with severe obesity and a GWAS study reported a single nucleotide polymorphism in RORα (rs7164773) was associated with increased risk for type 2 diabetes in the Mexico Mestizo population. Although we did not observe a statistically significant association between *RORA* expression and changes in metabolic factors among the adolescents, the suppression of *RORA* in late sleepers could signal circadian disruption that over time contributes to metabolic dysregulation.

Our findings highlight *NR1D2* (which encodes Rev-erb proteins) as potential mechanistic links between circadian disruption and dysregulation of glucose metabolism since they were connected both to late sleep timing and cardiometabolic outcomes. *CRY1* is another potential candidate, although its association with sleep midpoint was statistically significant only in the unadjusted model. The observed negative associations between the expression of *NR1D2* and *CRY1* with fasting glucose among the adolescents was consistent with mechanistic evidence in mice models. Rev-erbα/β (encoded by *NR1D1* and *NR1D2*, respectively) act as transcriptional repressors of many metabolic genes; for instance, Rev-erbα directly suppresses the gluconeogenic enzymes phosphoenolpyruvate carboxykinase and glucose-6-phosphatase, thereby suppressing hepatic glucose production [14]. Disruption of Rev-erb were found to increase glucose output and perturbs insulin signaling [38], while Rev-erb agonists were found to improve hyperglycemia [14]. Likewise, *CRY*-deficient mice exhibit elevated blood glucose and impaired glucose clearance during a glucose challenge [39]. Therefore, earlier sleepers with higher morning *CRY1* and *NR1D2* expression may have more robust or phase-appropriate clock repression of glucose-generating pathways, resulting in better glycemic control over time.

In this adolescent cohort, we observed notable sex-specific associations in circadian and metabolic outcomes. Specifically, the relationships between sleep midpoint and social jetlag with core clock gene expression were stronger in girls, as were associations between core clock gene expression and blood glucose. Conversely, associations of circadian gene expression with changes in blood cholesterol levels were more pronounced in boys. Several biological mechanisms might explain these sex-specific differences. One plausible factor is the influence of sex hormones. For example, estrogen signaling can directly alter the expression of several core clock genes and even shift circadian phase in animal models [40]. A recent study reported sex-specific gene expression rhythms, with women exhibiting a greater number of rhythmic transcripts compared to men, particularly in metabolically active tissues such as the liver [41]. Thus, the higher estrogen environment in post-pubertal girls may interact with circadian disruptions, intensifying circadian phase shifts and potentially rendering girls more sensitive to the perturbations in clock gene expression associated with late sleep timing.

Additionally, the significant negative associations of several core clock gene expression with changes in glucose levels observed specifically in girls may reflect a stronger underlying relationship between delayed sleep midpoint and insulin resistance previously reported in this adolescent cohort [20]. Sex differences in lipid metabolism may also contribute to the distinct strength of associations, between circadian gene expression and cholesterol changes, observed between boys and girls. For example, adiponectin concentrations, which are significantly higher in females compared to males, display a sexual dimorphism that emerges during puberty and relates closely to serum androgen levels [42]. Adiponectin plays a critical role in lipid metabolism by enhancing HDL cholesterol levels and promoting fatty acid oxidation, potentially leading to improved LDL cholesterol profiles [43]. Furthermore, recent evidence has shown that nearly all enzymes involved in cholesterol synthesis exhibit rhythmic expression in the liver; notably, this rhythmicity was dampened or absent in males, suggesting inherent sex differences in circadian regulation of cholesterol metabolism [41]. Collectively, such metabolic differences could underpin the sex-specific associations we observed between circadian gene expression and cholesterol levels. Overall, our findings highlight sex-related differences as an important modifier of circadian-metabolic relationships. It is also important to note that although analyses adjusted for pubertal status, residual differences in pubertal timing or maturation trajectories among boys and girls may persist. These differences could contribute to the observed sex-specific patterns, suggesting that the findings may reflect maturational processes rather than biological sex per se. Future research should further explore sex-specific chronobiology, including the influence of menstrual cycle phases, sex hormone fluctuations, pubertal stages, and sex-specific behavioral factors, to better understand the differential translation of circadian gene expression patterns into metabolic risk in males and females.

### 4.1. Strengths and limitations

This study has several notable strengths, including the novelty of our study question in this under-researched adolescent population, objectively-measured sleep characteristics from wrist-worn actigraphy, standardized blood collection timing, unbiased quantification of all genes, including the core circadian transcriptome, use of RNA-sequencing, and longitudinal measurements of multiple cardiometabolic biomarkers with standardized assays as outcomes. We also acknowledge several important limitations. First, because DESeq2 does not allow standard pooling across multiply imputed datasets, inference was based on a single completed dataset derived from multiply imputed datasets. Although this approach may slightly underestimate uncertainty, missingness was minimal and limited to covariates. Second, circadian gene expression was measured at a single morning time point so we cannot directly capture the amplitude or precise phase of each individual's gene oscillations in relation to sleep midpoints. While we interpreted lower morning expression as indicative of a phase delay in late sleepers, we did not directly measure each participant's circadian phase using validated markers such as dim-light melatonin onset or multiple time-point sampling. Third, gene expression was assayed from peripheral whole blood. Although circulating leucocytes can mirror systemic cues, they may not fully capture clock gene dynamics in metabolically critical organs, such as liver, adipose, or muscle. Fourth, our sample size (n = 203) limits our statistical power for detecting interaction effects, and some sex-specific analyses may have been underpowered. Thus, null findings in sex-stratified analyses should be interpreted with caution. Likewise, the sample size limited the statistical power to formally test whether circadian gene expression mediates the relationship between circadian misalignment and metabolic health, which remains an important avenue for future research in larger cohorts. Although pubertal status was adjusted for, pubertal maturation, sleep patterns, and metabolic regulation are closely intertwined during adolescence. Limited representation of earlier pubertal stages (<30% of the sample) in our study population limited our ability to test pubertal-stage–specific associations or interaction effects. Larger studies with more detailed pubertal staging are needed to clarify these potential interaction effects. Moreover, our follow-up period (~2 years) captures medium-term changes during adolescence; longer follow-up extending into adulthood would provide valuable information on whether these early observed alterations translate into clinically significant outcomes (e.g. metabolic syndrome, diabetes, or cardiovascular disease). Additionally, although our analytic sample (participants with available RNA-sequencing data) was slightly younger and more likely in earlier pubertal stages than those who do not have RNA-sequencing data, which may introduce selection bias, most socio-demographic and lifestyle factors were similarly distributed. Caution is warranted when generalizing our findings to broader adolescent population. Finally, although we adjusted for many potential confounders, and capitalized on a longitudinal cohort design, causal inference from our findings remains limited due to the observational nature of our study. Residual confounding from unmeasured factors likely persists, including chronotype and other biological traits related to circadian regulation, such as genetic differences in sensitivity to light, which we were unable to directly examine. Future research should incorporate direct assessments of chronotype alongside genetic data and objective measures of light exposure or light sensitivity to clarify how genetic predisposition and environmental factors jointly shape relationships between sleep timing and metabolic health in adolescents.

## 5. Conclusion

In summary, our findings provide novel clues about biological mechanisms that could link circadian disruption with cardiometabolic risk during the critical developmental window of adolescence. We found that later sleep midpoints among adolescents were associated with reduced morning expression levels of several core clock genes. Additionally, several of these genes were linked to changes in glucose and lipid metabolism biomarkers over the next two-year period, with sex-specific patterns observed. Future research incorporating multi-tissue omics approaches, formal mediation analyses, controlled experimental designs, and extended follow-up periods is needed to clarify causal pathways and inform targeted interventions for circadian and metabolic health. In the meantime, public health efforts aimed at promoting circadian hygiene and regular sleep timing in adolescents—using sex-specific or even personalized strategies—may offer a promising avenue for improving metabolic outcome later in life, with our findings offering a potential mechanistic support for such strategies. Furthermore, our results add to the growing body of evidence supporting later school start times for children, which have been shown to increase sleep duration and enhance circadian alignment. Overall, our findings underscore the likely importance of circadian health as a key pillar of cardiometabolic health, highlighting that *when* adolescents sleep matters for their metabolic health—beyond just *how much* they sleep.

## Supplementary Material

Supplemental Tables

## Figures and Tables

**Table 1a:** The characteristics of study participants by sleep midpoint.

Characteristics		Sleep Midpoint		*P* value
	Total	<4am	≥4am	
	N = 203	N = 124	N = 79	
Age, median (interquartile range)	13.6 (12.1-15.6)	13.2 (11.7-14.2)	14.3 (13.1-16.5)	<0.001
Sex, n(%)				0.29
male	96 (47.3%)	55 (44.4%)	41 (51.9%)	
female	107 (52.7%)	69 (55.6%)	38 (48.1%)	
Puberty stages, n(%)				0.003
earlier	51 (25.1%)	40 (32.3%)	11 (13.9%)	
later	152 (74.9%)	84 (67.7%)	68 (86.1%)	
Ever tried smoking, n(%)	34 (16.8%)	13 (10.5%)	21 (26.9%)	0.002
Ever drink alcohol or use substance, n(%)	150 (74.3%)	89 (71.8%)	61 (78.2%)	0.31
Sedentary time, mins/day	632.7 ± 77.1	626.6 ± 74.3	643.8 ± 81.7	0.18
Moderate and vigorous activities, mins/day	80.0 ± 28.8	83.9 ± 30.2	73.9 ± 25.5	0.017
Education level of head of the household, n(%)				0.32
less than high school	159 (78.3%)	100 (80.6%)	59 (74.7%)	
some high school and undergraduate	43 (21.2%)	24 (19.4%)	19 (24.1%)	
undergraduate degree or above	1 (0.5%)	0 (0.0%)	1 (1.3%)	
Socioeconomic status, n(%)				0.12
A/B	15 (7.4%)	11 (8.9%)	4 (5.1%)	
C	55 (27.1%)	29 (23.4%)	26 (32.9%)	
C+	34 (16.7%)	18 (14.5%)	16 (20.3%)	
D	9 (4.4%)	5 (4.0%)	4 (5.1%)	
D+	66 (32.5%)	41 (33.1%)	25 (31.6%)	
E	24 (11.8%)	20 (16.1%)	4 (5.1%)	
Weekday sleep duration, hours	8.5 ± 1.2	8.1 ± 1.1	9.1 ± 1.1	<0.001
InBody assessment time^2^, clock time	9:21:22 ± 0.7	9:22:27 ± 0.7	09:20:09 ± 0.6	0.34

**Table 1b:** The characteristics of study participants by social jetlag.

		Social Jetlag		*P* value
	Total	Jetlag < 2hrs	Jetlag ≥ 2hrs	
				
	N = 203	N = 167	N = 36	
Age, median (interquartile range)	13.6 (12.1-15.6)	13.6 (12.1-15.5)	13.8 (12.0-16.0)	0.75
Sex, n(%)				0.72
male	96 (47.3%)	78 (46.7%)	18 (50.0%)	
female	107 (52.7%)	89 (53.3%)	18 (50.0%)	
Puberty stages, n(%)				0.21
earlier	51 (25.1%)	39 (23.4%)	12 (33.3%)	
later	152 (74.9%)	128 (76.6%)	24 (66.7%)	
Ever smoke snuff, n(%)	34 (16.8%)	29 (17.5%)	5 (13.9%)	0.60
Ever drink alcohol or use substance, n(%)	150 (74.3%)	121 (72.9%)	29 (80.6%)	0.34
Sedentary time, mins/day	632.7 ± 77.1	629.3 ± 78.7	646.0 ± 69.9	0.28
Moderate and vigorous activities, mins/day	80.0 ± 28.8	81.4 ± 29.1	73.0 ± 26.2	0.12
Education level of head of the household, n(%)				0.86
less than high school	159 (78.3%)	130 (77.8%)	29 (80.6%)	
some high school and undergraduate	43 (21.2%)	36 (21.6%)	7 (19.4%)	
undergraduate degree or above	1 (0.5%)	1 (0.6%)	0 (0.0%)	
Socioeconomic status, n(%)				0.11
A/B	15 (7.4%)	13 (7.8%)	2 (5.6%)	
C	55 (27.1%)	41 (24.6%)	14 (38.9%)	
C+	34 (16.7%)	30 (18.0%)	4 (11.1%)	
D	9 (4.4%)	5 (3.0%)	4 (11.1%)	
D+	66 (32.5%)	57 (34.1%)	9 (25.0%)	
E	24 (11.8%)	21 (12.6%)	3 (8.3%)	
Weekday sleep duration, hours	8.5 ± 1.2	8.7 ± 1.1	7.5 ± 1.1	<0.001
InBody assessment time and ^2^, clock time	9:21:22 ± 0.7	9:25:27 ± 0.7	9:34:17 ± 0.8	0.24

1 Data are presented as mean ± SD or n (%), unless otherwise indicated.

2 InBody assessment time approximates when blood samples were drawn.

**Table 2:** Association between sleep midpoint and social jetlag with morning core clock gene expression.

Gene	Sleep midpoint		Social jetlag	
	β^a^	*P* value	β	*P* value
*CRY1*	−0.115	0.063	0.113	0.234
*PER3*	−0.116	0.109	0.194	0.077
*RORA*	**−0.190** ^b^	**0.001**	0.080	0.355
*CRY2*	−0.054	0.086	0.058	0.236
*NR1D1*	−0.078	0.059	0.052	0.414
*PER2*	−0.091	0.188	0.141	0.181
*BMAL1*	−0.066	0.153	0.038	0.595
*CLOCK*	**−0.141**	**0.019**	0.021	0.818
*RORC*	**−0.147**	**0.039**	0.152	0.165
*NR1D2*	**−0.093**	**0.029**	0.091	0.162
*PER1*	0.007	0.913	0.090	0.312

a β represents the log2 fold change in gene expression per unit increase in sleep midpoint or social jetlag. Models are adjusted for age, sex, puberty onset, smoking status, drinking status, sedentary time, moderate to vigorous physical activity time, education level of head of the household, socioeconomic status, weekday sleep duration, and InBody assessment time.

b Bolded values are statistically significant at alpha level of 0.05.

**Table 3:** Sex stratified associations of sleep midpoint and social jetlag with morning gene expression levels.

Gene	Sleep midpoint				Social jetlag			
	Female		Male		Female		Male	
	β^a^	*P* value	β	*P* value	β	*P* value	β	*P* value
*CRY1*	−0.132	0.140	−0.074	0.414	0.097	0.504	0.136	0.310
*PER3*	−0.172	0.070	−0.049	0.678	0.240	0.114	0.230	0.182
*RORA*	**−0.255** ^b^	**0.002**	−0.124	0.086	0.198	0.142	−0.019	0.857
*CRY2*	−0.067	0.132	−0.033	0.495	0.091	0.203	0.027	0.711
*NR1D1*	−0.074	0.221	−0.091	0.139	0.103	0.288	0.031	0.735
*PER2*	−0.101	0.249	−0.103	0.377	0.244	0.080	0.068	0.697
*BMAL1*	−0.095	0.162	−0.020	0.762	0.059	0.593	0.023	0.814
*CLOCK*	−0.155	0.089	−0.112	0.179	0.035	0.813	0.040	0.748
*RORC*	−0.154	0.066	−0.186	0.122	0.124	0.360	0.094	0.601
*NR1D2*	−0.117	0.071	−0.081	0.144	0.176	0.090	0.021	0.805
*PER1*	−0.069	0.349	0.123	0.215	**0.275**	**0.017**	−0.214	0.140

a β represents the log2 fold change in gene expression per unit increase in sleep midpoint or social jetlag. Models are adjusted for age, puberty onset, smoking status, drinking status, sedentary time, moderate to vigorous physical activity time, education level of head of the household, socioeconomic status, weekday sleep duration, and InBody assessment time.

b Bolded values are statistically significant at alpha level of 0.05.

## Data Availability

The data underlying this article will be shared on reasonable request to the corresponding author. Some of the data are available through the National Institutes of Health Human Health Exposure Analysis Resource (NIH HHEAR) data repository (dois: 10.36043/1431_392, 10.36043/1431_327).

## Appendix A. Supplementary data

Supplementary data to this article can be found online at https://doi.org/10.1016/j.sleep.2026.108819.
