# Supplementary material for: Circadian gene expression in adolescents: Associations with concurrent circadian disruption and subsequent changes in cardiometabolic risk measures
Source: Sleep Med. Author manuscript; Available in PMC 2026 Jun 14. (PMC13265021; doi:10.1016/j.sleep.2026.108819)
Supplement: Supplemental Tables [file NIHMS2179872-supplement-Supplemental_Tables.docx]

Adolescents amid pubertal transition recruited at T1 visit in 2015

(n=554)

RNA-sequencing data passed quality control and included in the analysis of sleep midpoint and core clock gene expression

(n=203)

Anthropometric outcome measurements (BMI, WC, SBP, and DBP) available at both T1 and T2 visit in 2017

(n=190)

Blood measured risk factors (glucose, HOMA-IR, insulin, and cholesterol) available at both T1 and T2 visit in 2017

(n=165)

**Supplement Figure 1: Flowchart for inclusion in the analytic sample of participants in the ELEMENT Cohort.**

Abbreviations: BMI, body mass index; WC, waist circumference; SBP, Systolic blood pressure; DBP. diastolic blood pressure; HOMA-IR, Homeostatic Model Assessment of Insulin Resistance


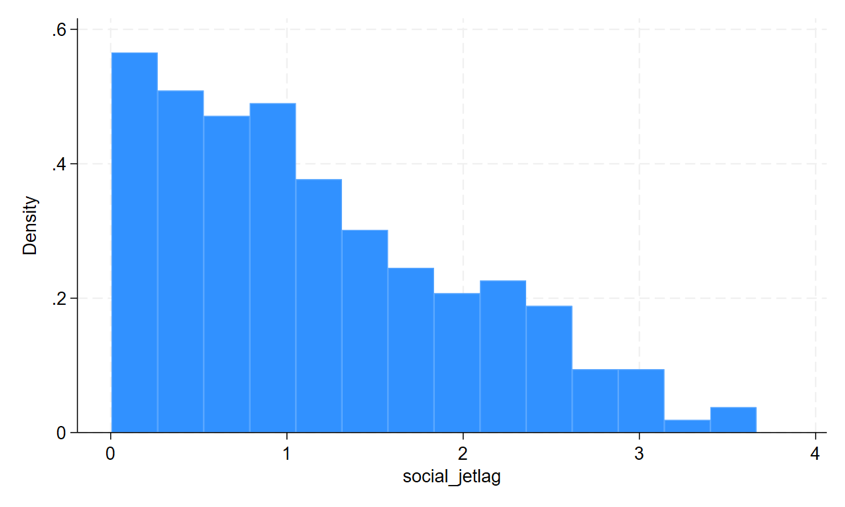


**Supplemental Figure 2-1. Distribution of social jetlag (absolute values).**


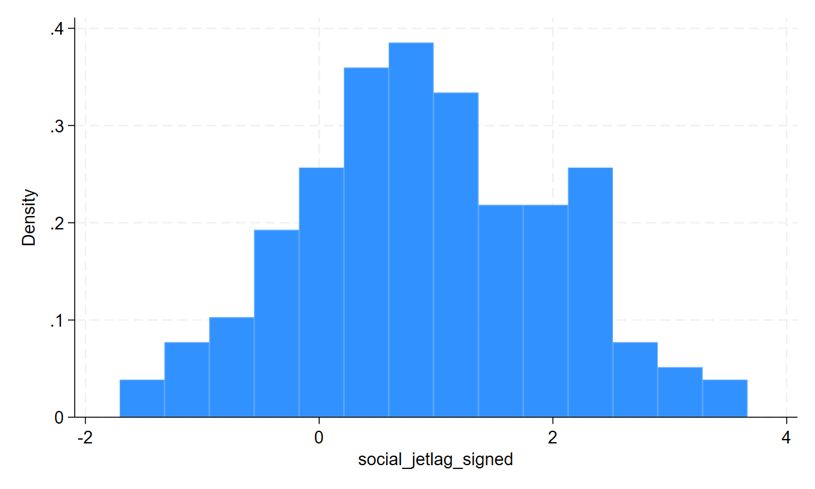


**Supplemental Figure 2-2. Distribution of signed social jetlag.**


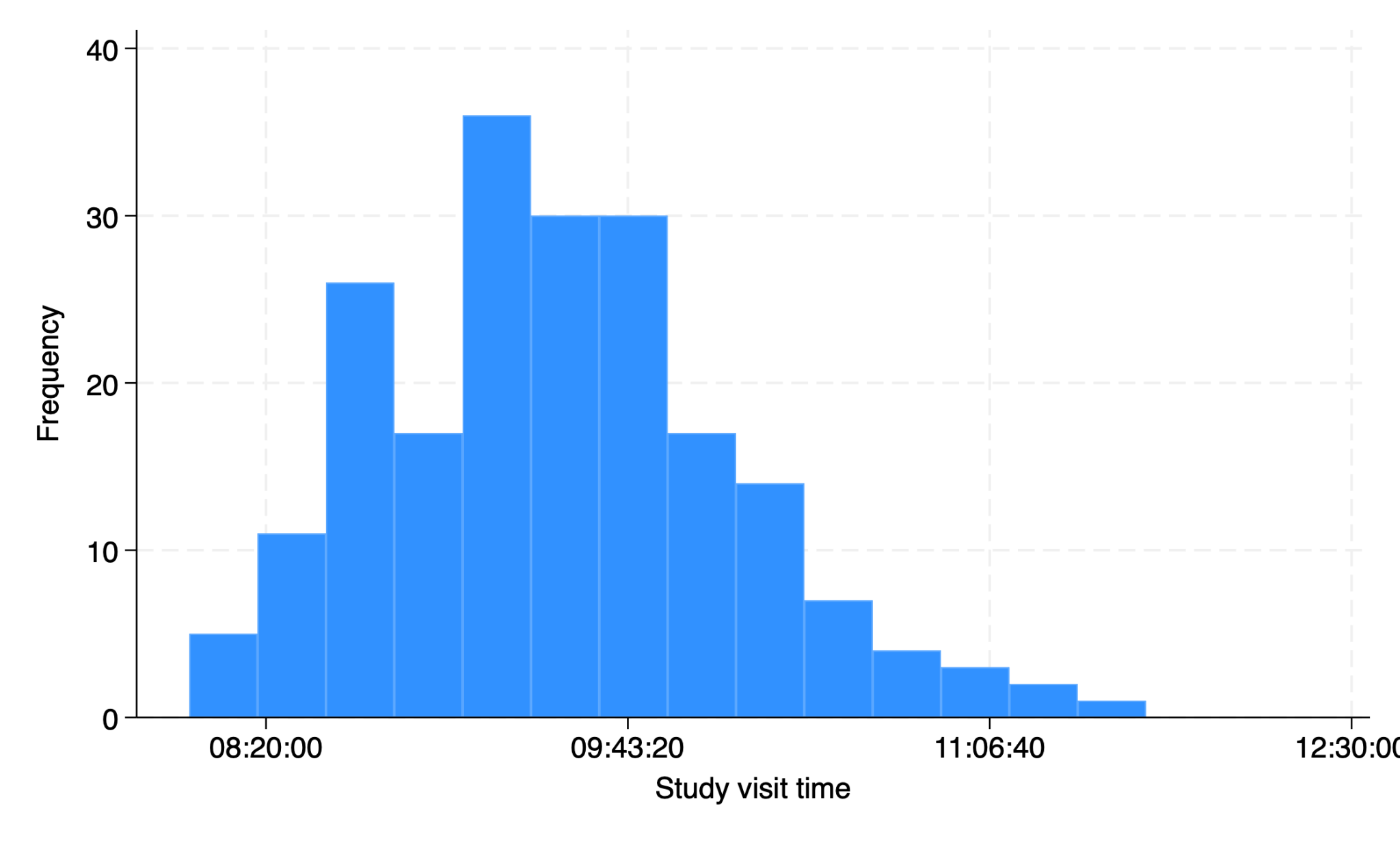


**Supplemental Figure 3. The distribution of InBody assessment time (approximates when blood samples were drawn).**


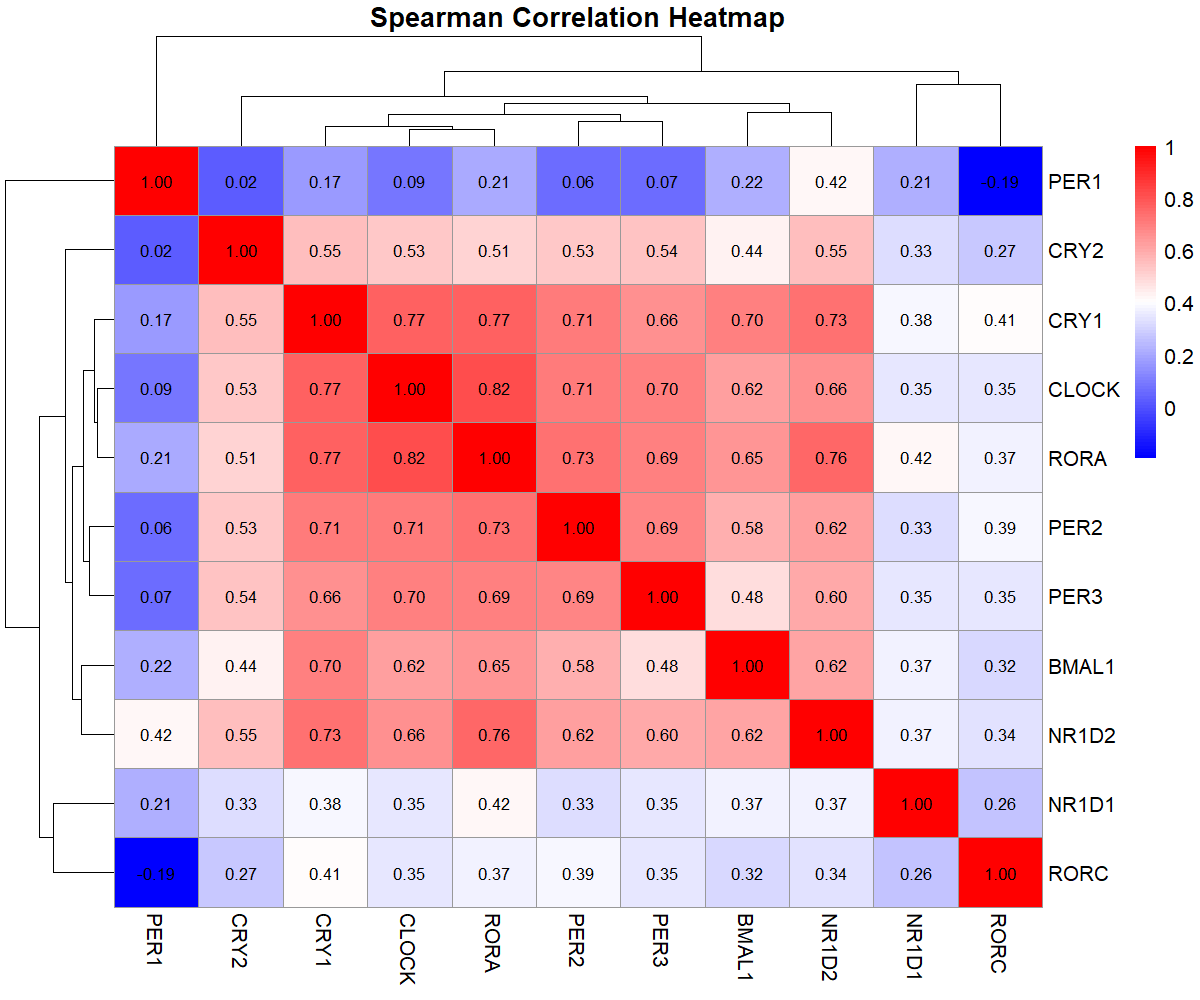


**Supplemental Figure 4. Spearman’s rank correlation coefficients among the core clock genes expression levels.**

**Supplemental Table 1. Associations between sleep midpoint and social jetlag with core clock gene expression levels (adjusted for month of study visit at T1 in addition to covariates in the main analysis).**

|  | Sleep midpoint | | Social jetlag | |
| --- | --- | --- | --- | --- |
| Gene | β^1^ | *P* value | β | *P* value |
| *CRY1* | -0.101 | 0.112 | 0.099 | 0.315 |
| *PER3* | -0.116 | 0.122 | 0.183 | 0.110 |
| *RORA* | **-0.177**^2^ | **0.001** | 0.090 | 0.295 |
| *CRY2* | -0.041 | 0.207 | 0.046 | 0.360 |
| *NR1D1* | -0.068 | 0.091 | 0.045 | 0.475 |
| *PER2* | -0.093 | 0.183 | 0.151 | 0.162 |
| *BMAL1* | -0.052 | 0.270 | 0.026 | 0.723 |
| *CLOCK* | **-0.124** | **0.044** | -0.002 | 0.983 |
| *RORC* | **-0.148** | **0.044** | 0.151 | 0.184 |
| *NR1D2* | -0.076 | 0.080 | 0.085 | 0.199 |
| *PER1* | 0.012 | 0.835 | 0.032 | 0.705 |

^1^Beta represents the log2fold change in gene expression per unit increase in sleep midpoint or social jetlag. Models are adjusted for age, sex, puberty onset, smoking status, drinking status, sedentary time, moderate to vigorous physical activity time, education level of head of the household, socioeconomic status, weekday sleep duration, InBody assessment time, and month of the study visit. ^2 2^Bolded values are statistically significant at alpha level of 0.05.

**Supplemental Table 2. Association between sleep midpoint and social jetlag with morning core clock gene expression, additionally adjusted for neutrophils and eosinophils**.

|  | Sleep midpoint | | Social jetlag | |
| --- | --- | --- | --- | --- |
| Gene | β^1^ | *P* value | β | *P* value |
| *CRY1* | -0.104 | 0.0883 | 0.113 | 0.2253 |
| *PER3* | -0.094 | 0.1895 | 0.190 | 0.0820 |
| *RORA* | **-0.168** | **0.0019** | 0.072 | 0.3921 |
| *CRY2* | -0.045 | 0.1471 | 0.051 | 0.2868 |
| *NR1D1* | -0.076 | 0.0742 | 0.047 | 0.4742 |
| *PER2* | -0.083 | 0.2136 | 0.160 | 0.1162 |
| *BMAL1* | -0.063 | 0.1663 | 0.054 | 0.4367 |
| *CLOCK* | **-0.122** | **0.0427** | 0.011 | 0.9010 |
| *RORC* | -0.138 | 0.0535 | 0.163 | 0.1359 |
| *NR1D2* | **-0.080** | **0.0497** | 0.081 | 0.1935 |
| *PER1* | 0.011 | 0.8463 | 0.061 | 0.4925 |

^1^β represents the log2 fold change in gene expression per unit increase in sleep midpoint or social jetlag. Models are adjusted for age, sex, puberty onset, smoking status, drinking status, sedentary time, moderate to vigorous physical activity time, education level of head of the household, socioeconomic status, weekday sleep duration, InBody assessment time, proportion of neutrophils, and proportion of eosinophils. ^2^Bolded values are statistically significant at alpha level of 0.05.

**Supplemental Table 3. Unadjusted associations between sleep midpoint and social jetlag with core clock gene expression levels.**

|  | **Sleep midpoint** |  | **Social jetlag** | |
| --- | --- | --- | --- | --- |
| Gene | β^1^ | *P* value | β | *P* value |
| *CRY1* | **-0.132** | **0.013** | 0.118 | 0.155 |
| *PER3* | -0.089 | 0.143 | 0.181 | 0.055 |
| *RORA* | **-0.186** | **0.000** | 0.074 | 0.339 |
| *CRY2* | **-0.059** | **0.033** | 0.068 | 0.115 |
| *NR1D1* | -0.059 | 0.095 | 0.049 | 0.369 |
| *PER2* | -0.073 | 0.202 | 0.113 | 0.207 |
| *BMAL1* | -0.066 | 0.089 | 0.046 | 0.445 |
| *CLOCK* | **-0.140** | **0.006** | 0.031 | 0.697 |
| *RORC* | **-0.160** | **0.007** | **0.199** | **0.033** |
| *NR1D2* | **-0.089** | **0.022** | 0.097 | 0.107 |
| *PER1* | 0.102 | 0.063 | 0.014 | 0.868 |

^1^ Beta represents the log2fold change in gene expression per unit increase in sleep midpoint or social jetlag. ^2^ Bolded values are statistically significant at alpha level of 0.05.

**Supplemental Table 4. Association between social jetlag (signed) and morning core clock gene expression.**

| Gene | β^1^ | *P* value |
| --- | --- | --- |
| *CRY1* | 0.05 | 0.519 |
| *PER3* | 0.09 | 0.353 |
| *RORA* | 0.05 | 0.516 |
| *CRY2* | 0.03 | 0.528 |
| *NR1D1* | 0.02 | 0.690 |
| *PER2* | 0.06 | 0.533 |
| *BMAL1* | -0.01 | 0.885 |
| *CLOCK* | -0.03 | 0.680 |
| *RORC* | 0.12 | 0.195 |
| *NR1D2* | 0.05 | 0.368 |
| *PER1* | **0.20** | **0.007** |

^1^β represents the log2 fold change in gene expression per unit increase in sleep midpoint or social jetlag. Models are adjusted for age, sex, puberty onset, smoking status, drinking status, sedentary time, moderate to vigorous physical activity time, education level of head of the household, socioeconomic status, weekday sleep duration, and InBody assessment time. ^2^Bolded values are statistically significant at alpha level of 0.05.

**Supplemental Table 5. Associations between core clock gene expression and changes in metabolic biomarkers.**

| Outcome^1^ | Gene | Effect Size^2^ | *P* Value |
| --- | --- | --- | --- |
| BMI | *BMAL1* | 0.0000 | 0.969 |
| BMI | *CLOCK* | -0.0002 | 0.754 |
| BMI | *CRY1* | -0.0004 | 0.629 |
| BMI | *CRY2* | 0.0000 | 0.955 |
| BMI | *NR1D1* | -0.0005 | 0.471 |
| BMI | *NR1D2* | -0.0002 | 0.583 |
| BMI | *PER1* | 0.0000 | 0.564 |
| BMI | *PER2* | -0.0002 | 0.873 |
| BMI | *PER3* | 0.0003 | 0.809 |
| BMI | *RORA* | 0.0000 | 0.773 |
| BMI | *RORC* | 0.0005 | 0.527 |
| Waist circumference | *BMAL1* | 0.0000 | 0.948 |
| Waist circumference | *CLOCK* | -0.0001 | 0.893 |
| Waist circumference | *CRY1* | -0.0003 | 0.674 |
| Waist circumference | *CRY2* | 0.0004 | 0.544 |
| Waist circumference | *NR1D1* | -0.0002 | 0.738 |
| Waist circumference | *NR1D2* | -0.0003 | 0.505 |
| Waist circumference | *PER1* | 0.0000 | 0.585 |
| Waist circumference | *PER2* | 0.0008 | 0.427 |
| Waist circumference | *PER3* | 0.0008 | 0.564 |
| Waist circumference | *RORA* | 0.0000 | 0.908 |
| Waist circumference | *RORC* | 0.0010 | 0.226 |
| SBP | *BMAL1* | -0.0004 | 0.442 |
| SBP | *CLOCK* | -0.0005 | 0.477 |
| SBP | *CRY1* | 0.0001 | 0.943 |
| SBP | *CRY2* | 0.0000 | 0.942 |
| SBP | *NR1D1* | 0.0003 | 0.639 |
| SBP | *NR1D2* | 0.0000 | 0.971 |
| SBP | *PER1* | 0.0000 | 0.962 |
| SBP | *PER2* | 0.0007 | 0.515 |
| SBP | *PER3* | -0.0010 | 0.481 |
| SBP | *RORA* | 0.0000 | 0.984 |
| SBP | *RORC* | -0.0002 | 0.770 |
| DBP | *BMAL1* | -0.0001 | 0.810 |
| DBP | *CLOCK* | -0.0005 | 0.472 |
| DBP | *CRY1* | 0.0002 | 0.832 |
| DBP | *CRY2* | -0.0002 | 0.798 |
| DBP | *NR1D1* | 0.0013 | 0.056 |
| DBP | *NR1D2* | 0.0001 | 0.848 |
| DBP | *PER1* | 0.0001 | 0.135 |
| DBP | *PER2* | 0.0003 | 0.785 |
| DBP | *PER3* | -0.0016 | 0.255 |
| DBP | *RORA* | 0.0000 | 0.973 |
| DBP | *RORC* | -0.0001 | 0.870 |
| Glucose | *BMAL1* | 0.0001 | 0.861 |
| Glucose | *CLOCK* | -0.0012 | 0.113 |
| Glucose | *CRY1* | -0.0018 | 0.046 |
| Glucose | *CRY2* | -0.0012 | 0.059 |
| Glucose | *NR1D1* | -0.0001 | 0.857 |
| Glucose | *NR1D2* | -0.0010 | 0.017 |
| Glucose | *PER1* | -0.0001 | 0.111 |
| Glucose | *PER2* | -0.0012 | 0.279 |
| Glucose | *PER3* | -0.0020 | 0.192 |
| Glucose | *RORA* | -0.0002 | 0.170 |
| Glucose | *RORC* | -0.0004 | 0.643 |
| HOMA-IR | *BMAL1* | 0.0005 | 0.357 |
| HOMA-IR | *CLOCK* | 0.0007 | 0.364 |
| HOMA-IR | *CRY1* | 0.0001 | 0.942 |
| HOMA-IR | *CRY2* | 0.0004 | 0.556 |
| HOMA-IR | *NR1D1* | 0.0008 | 0.334 |
| HOMA-IR | *NR1D2* | -0.0001 | 0.774 |
| HOMA-IR | *PER1* | 0.0000 | 0.231 |
| HOMA-IR | *PER2* | 0.0007 | 0.524 |
| HOMA-IR | *PER3* | 0.0005 | 0.747 |
| HOMA-IR | *RORA* | 0.0001 | 0.455 |
| HOMA-IR | *RORC* | -0.0005 | 0.620 |
| Insulin | *BMAL1* | 0.0005 | 0.360 |
| Insulin | *CLOCK* | 0.0009 | 0.294 |
| Insulin | *CRY1* | 0.0001 | 0.949 |
| Insulin | *CRY2* | 0.0001 | 0.928 |
| Insulin | *NR1D1* | 0.0010 | 0.221 |
| Insulin | *NR1D2* | -0.0003 | 0.495 |
| Insulin | *PER1* | 0.0000 | 0.273 |
| Insulin | *PER2* | 0.0003 | 0.812 |
| Insulin | *PER3* | 0.0003 | 0.836 |
| Insulin | *RORA* | 0.0001 | 0.414 |
| Insulin | *RORC* | -0.0003 | 0.778 |
| Total cholesterol | *BMAL1* | 0.0010 | 0.048 |
| Total cholesterol | *CLOCK* | 0.0008 | 0.260 |
| Total cholesterol | *CRY1* | 0.0018 | 0.039 |
| Total cholesterol | *CRY2* | 0.0005 | 0.438 |
| Total cholesterol | *NR1D1* | 0.0000 | 0.997 |
| Total cholesterol | *NR1D2* | 0.0004 | 0.305 |
| Total cholesterol | *PER1* | 0.0001 | 0.127 |
| Total cholesterol | *PER2* | 0.0014 | 0.175 |
| Total cholesterol | *PER3* | 0.0037 | 0.011 |
| Total cholesterol | *RORA* | 0.0001 | 0.355 |
| Total cholesterol | *RORC* | -0.0001 | 0.880 |
| HDL cholesterol | *BMAL1* | 0.0002 | 0.727 |
| HDL cholesterol | *CLOCK* | 0.0004 | 0.644 |
| HDL cholesterol | *CRY1* | 0.0008 | 0.391 |
| HDL cholesterol | *CRY2* | 0.0001 | 0.816 |
| HDL cholesterol | *NR1D1* | 0.0003 | 0.661 |
| HDL cholesterol | *NR1D2* | 0.0002 | 0.625 |
| HDL cholesterol | *PER1* | 0.0000 | 0.500 |
| HDL cholesterol | *PER2* | -0.0001 | 0.906 |
| HDL cholesterol | *PER3* | 0.0013 | 0.385 |
| HDL cholesterol | *RORA* | 0.0001 | 0.656 |
| HDL cholesterol | *RORC* | -0.0004 | 0.651 |
| LDL cholesterol | *BMAL1* | 0.0011 | 0.019 |
| LDL cholesterol | *CLOCK* | 0.0009 | 0.200 |
| LDL cholesterol | *CRY1* | 0.0018 | 0.032 |
| LDL cholesterol | *CRY2* | 0.0001 | 0.894 |
| LDL cholesterol | *NR1D1* | 0.0003 | 0.647 |
| LDL cholesterol | *NR1D2* | 0.0005 | 0.185 |
| LDL cholesterol | *PER1* | 0.0001 | 0.044 |
| LDL cholesterol | *PER2* | 0.0015 | 0.133 |
| LDL cholesterol | *PER3* | 0.0029 | 0.043 |
| LDL cholesterol | *RORA* | 0.0002 | 0.212 |
| LDL cholesterol | *RORC* | -0.0003 | 0.711 |
| Triglycerides | *BMAL1* | 0.0002 | 0.717 |
| Triglycerides | *CLOCK* | -0.0002 | 0.751 |
| Triglycerides | *CRY1* | 0.0002 | 0.857 |
| Triglycerides | *CRY2* | 0.0002 | 0.779 |
| Triglycerides | *NR1D1* | -0.0013 | 0.104 |
| Triglycerides | *NR1D2* | -0.0001 | 0.781 |
| Triglycerides | *PER1* | 0.0001 | 0.147 |
| Triglycerides | *PER2* | 0.0004 | 0.741 |
| Triglycerides | *PER3* | 0.0011 | 0.489 |
| Triglycerides | *RORA* | 0.0000 | 0.792 |
| Triglycerides | *RORC* | 0.0005 | 0.569 |

^1^Outcome was standardized to have a mean of 0 and standard deviation of 1. ^2^Models are adjusted for age, sex, puberty onset, smoking status, drinking status, sedentary time, moderate to vigorous physical activity time, education level of head of the household, socioeconomic status, weekday sleep duration, and InBody assessment time. Models were additionally adjusted for height when using systolic or diastolic blood pressure as the outcomes.

**
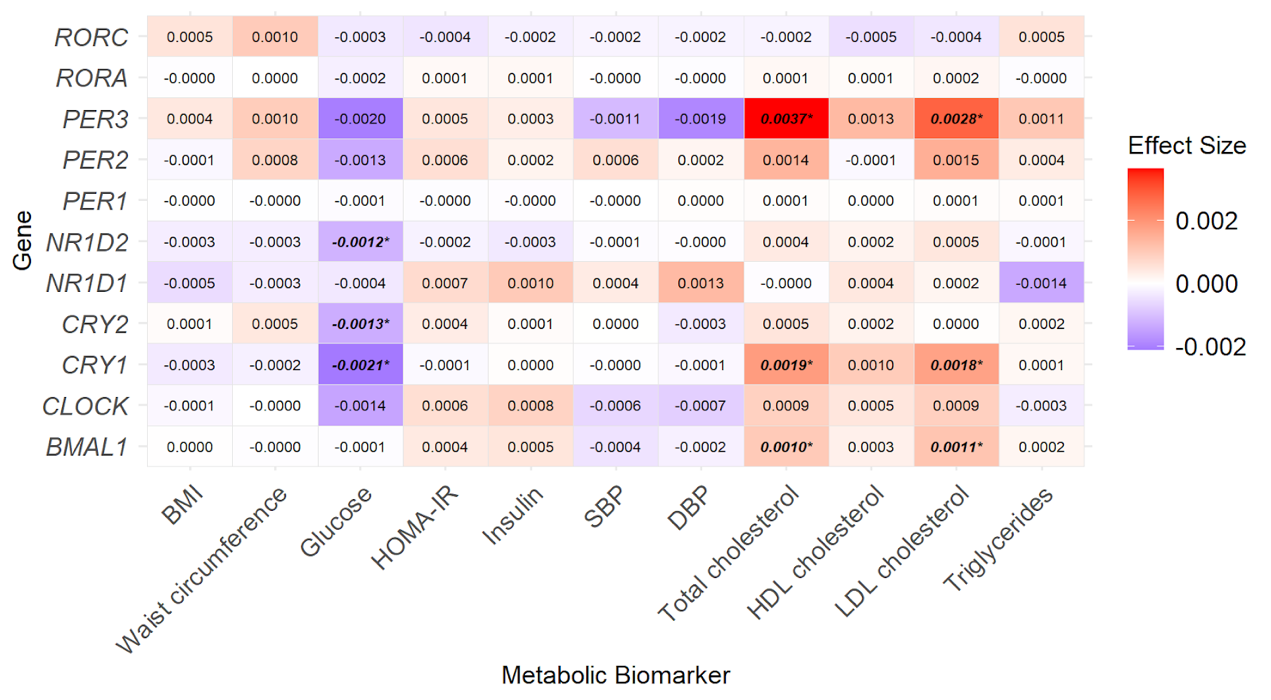
**

**Supplemental Figure 5. Associations between morning core clock gene expression and changes in metabolic biomarkers over a two-year period, additionally adjusting for dietary patterns.** Cardiometabolic risk factors were standardized to have a mean of 0 and standard deviation of 1. Models are adjusted for age, sex, puberty onset, smoking status, drinking status, sedentary time, moderate to vigorous physical activity time, education level of head of the household, socioeconomic status, weekday sleep duration, InBody assessment time, and dietary patterns. Models were additionally adjusted for height when using systolic or diastolic blood pressure as the outcomes. Effect sizes are represented by colors and color gradients. Results shown with * are statistically significant at alpha level of 0.05.


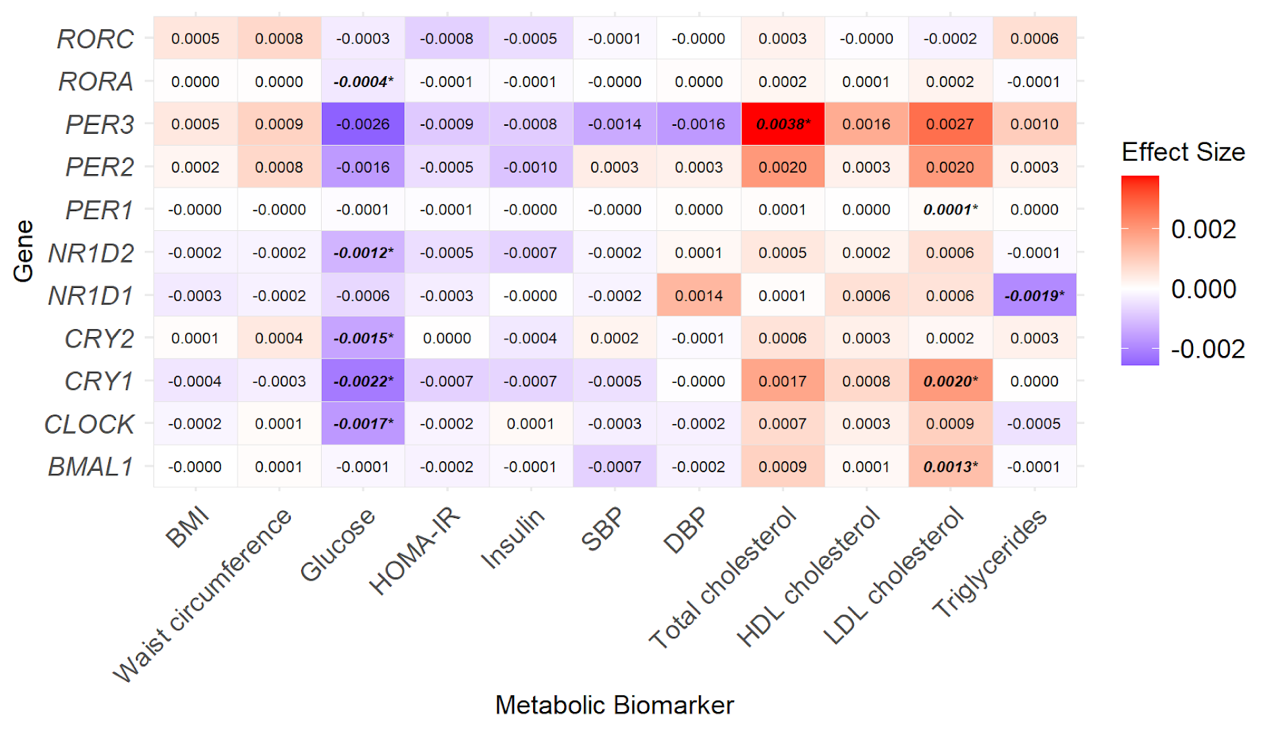


**Supplemental Figure 6. Associations between morning core clock gene expression and changes in metabolic biomarkers over a two-year period, additionally adjusting for month of the study visit.** Cardiometabolic risk factors were standardized to have a mean of 0 and standard deviation of 1. Models are adjusted for age, sex, puberty onset, smoking status, drinking status, sedentary time, moderate to vigorous physical activity time, education level of head of the household, socioeconomic status, weekday sleep duration, InBody assessment time, and month of the study visit. Models were additionally adjusted for height when using systolic or diastolic blood pressure as the outcomes. Effect sizes are represented by colors and color gradients. Results shown with * are statistically significant at alpha level of 0.05.

**Supplemental Table 6. Associations between core clock gene expression and changes in metabolic biomarkers among the females and males.**

| Outcome^1^ | Gene | Effect Size^2^ | *P* Value | sex |
| --- | --- | --- | --- | --- |
| BMI | *BMAL1* | 0.0006 | 0.431 | Male |
| BMI | *CLOCK* | 0.0007 | 0.528 | Male |
| BMI | *CRY1* | -0.0002 | 0.857 | Male |
| BMI | *CRY2* | 0.0009 | 0.352 | Male |
| BMI | *NR1D1* | 0.0011 | 0.329 | Male |
| BMI | *NR1D2* | 0.0009 | 0.238 | Male |
| BMI | *PER1* | 0.0000 | 0.504 | Male |
| BMI | *PER2* | 0.0005 | 0.761 | Male |
| BMI | *PER3* | 0.0007 | 0.738 | Male |
| BMI | *RORA* | 0.0002 | 0.436 | Male |
| BMI | *RORC* | 0.0023 | 0.042 | Male |
| Waist circumference | *BMAL1* | 0.0003 | 0.704 | Male |
| Waist circumference | *CLOCK* | 0.0005 | 0.651 | Male |
| Waist circumference | *CRY1* | -0.0010 | 0.486 | Male |
| Waist circumference | *CRY2* | 0.0014 | 0.171 | Male |
| Waist circumference | *NR1D1* | 0.0010 | 0.355 | Male |
| Waist circumference | *NR1D2* | 0.0006 | 0.464 | Male |
| Waist circumference | *PER1* | 0.0000 | 0.665 | Male |
| Waist circumference | *PER2* | 0.0005 | 0.744 | Male |
| Waist circumference | *PER3* | 0.0003 | 0.907 | Male |
| Waist circumference | *RORA* | 0.0001 | 0.636 | Male |
| Waist circumference | *RORC* | 0.0017 | 0.134 | Male |
| SBP | *BMAL1* | -0.0004 | 0.612 | Male |
| SBP | *CLOCK* | 0.0003 | 0.755 | Male |
| SBP | *CRY1* | 0.0008 | 0.529 | Male |
| SBP | *CRY2* | -0.0005 | 0.609 | Male |
| SBP | *NR1D1* | 0.0001 | 0.935 | Male |
| SBP | *NR1D2* | 0.0001 | 0.860 | Male |
| SBP | *PER1* | 0.0000 | 0.624 | Male |
| SBP | *PER2* | 0.0000 | 0.993 | Male |
| SBP | *PER3* | -0.0008 | 0.710 | Male |
| SBP | *RORA* | 0.0002 | 0.427 | Male |
| SBP | *RORC* | 0.0005 | 0.656 | Male |
| DBP | *BMAL1* | -0.0009 | 0.235 | Male |
| DBP | *CLOCK* | -0.0011 | 0.333 | Male |
| DBP | *CRY1* | 0.0003 | 0.807 | Male |
| DBP | *CRY2* | -0.0013 | 0.229 | Male |
| DBP | *NR1D1* | 0.0017 | 0.141 | Male |
| DBP | *NR1D2* | 0.0000 | 0.996 | Male |
| DBP | *PER1* | 0.0000 | 0.342 | Male |
| DBP | *PER2* | -0.0016 | 0.333 | Male |
| DBP | *PER3* | -0.0024 | 0.276 | Male |
| DBP | *RORA* | 0.0000 | 0.995 | Male |
| DBP | *RORC* | -0.0003 | 0.814 | Male |
| Glucose | *BMAL1* | 0.0008 | 0.403 | Male |
| Glucose | *CLOCK* | -0.0011 | 0.397 | Male |
| Glucose | *CRY1* | -0.0012 | 0.450 | Male |
| Glucose | *CRY2* | -0.0015 | 0.208 | Male |
| Glucose | *NR1D1* | -0.0001 | 0.948 | Male |
| Glucose | *NR1D2* | -0.0010 | 0.222 | Male |
| Glucose | *PER1* | 0.0000 | 0.944 | Male |
| Glucose | *PER2* | -0.0004 | 0.836 | Male |
| Glucose | *PER3* | -0.0011 | 0.657 | Male |
| Glucose | *RORA* | -0.0001 | 0.675 | Male |
| Glucose | *RORC* | -0.0012 | 0.377 | Male |
| HOMA-IR | *BMAL1* | 0.0017 | 0.042 | Male |
| HOMA-IR | *CLOCK* | 0.0016 | 0.177 | Male |
| HOMA-IR | *CRY1* | 0.0009 | 0.546 | Male |
| HOMA-IR | *CRY2* | 0.0007 | 0.507 | Male |
| HOMA-IR | *NR1D1* | 0.0013 | 0.255 | Male |
| HOMA-IR | *NR1D2* | 0.0009 | 0.228 | Male |
| HOMA-IR | *PER1* | 0.0000 | 0.844 | Male |
| HOMA-IR | *PER2* | 0.0019 | 0.268 | Male |
| HOMA-IR | *PER3* | 0.0035 | 0.132 | Male |
| HOMA-IR | *RORA* | 0.0003 | 0.266 | Male |
| HOMA-IR | *RORC* | -0.0001 | 0.933 | Male |
| Insulin | *BMAL1* | 0.0018 | 0.028 | Male |
| Insulin | *CLOCK* | 0.0020 | 0.099 | Male |
| Insulin | *CRY1* | 0.0008 | 0.583 | Male |
| Insulin | *CRY2* | 0.0012 | 0.256 | Male |
| Insulin | *NR1D1* | 0.0016 | 0.178 | Male |
| Insulin | *NR1D2* | 0.0011 | 0.167 | Male |
| Insulin | *PER1* | 0.0000 | 0.858 | Male |
| Insulin | *PER2* | 0.0017 | 0.325 | Male |
| Insulin | *PER3* | 0.0035 | 0.123 | Male |
| Insulin | *RORA* | 0.0003 | 0.238 | Male |
| Insulin | *RORC* | 0.0001 | 0.950 | Male |
| Total cholesterol | *BMAL1* | 0.0016 | 0.039 | Male |
| Total cholesterol | *CLOCK* | 0.0005 | 0.679 | Male |
| Total cholesterol | *CRY1* | 0.0031 | 0.026 | Male |
| Total cholesterol | *CRY2* | 0.0002 | 0.842 | Male |
| Total cholesterol | *NR1D1* | 0.0010 | 0.358 | Male |
| Total cholesterol | *NR1D2* | 0.0016 | 0.028 | Male |
| Total cholesterol | *PER1* | 0.0001 | 0.076 | Male |
| Total cholesterol | *PER2* | 0.0023 | 0.165 | Male |
| Total cholesterol | *PER3* | 0.0056 | 0.010 | Male |
| Total cholesterol | *RORA* | 0.0003 | 0.243 | Male |
| Total cholesterol | *RORC* | 0.0001 | 0.959 | Male |
| HDL cholesterol | *BMAL1* | 0.0000 | 0.979 | Male |
| HDL cholesterol | *CLOCK* | -0.0007 | 0.540 | Male |
| HDL cholesterol | *CRY1* | 0.0015 | 0.312 | Male |
| HDL cholesterol | *CRY2* | -0.0010 | 0.376 | Male |
| HDL cholesterol | *NR1D1* | 0.0010 | 0.373 | Male |
| HDL cholesterol | *NR1D2* | 0.0000 | 0.965 | Male |
| HDL cholesterol | *PER1* | 0.0000 | 0.614 | Male |
| HDL cholesterol | *PER2* | -0.0013 | 0.431 | Male |
| HDL cholesterol | *PER3* | 0.0006 | 0.792 | Male |
| HDL cholesterol | *RORA* | -0.0001 | 0.831 | Male |
| HDL cholesterol | *RORC* | -0.0009 | 0.431 | Male |
| LDL cholesterol | *BMAL1* | 0.0022 | 0.005 | Male |
| LDL cholesterol | *CLOCK* | 0.0012 | 0.289 | Male |
| LDL cholesterol | *CRY1* | 0.0034 | 0.014 | Male |
| LDL cholesterol | *CRY2* | 0.0006 | 0.551 | Male |
| LDL cholesterol | *NR1D1* | 0.0010 | 0.356 | Male |
| LDL cholesterol | *NR1D2* | 0.0023 | 0.002 | Male |
| LDL cholesterol | *PER1* | 0.0001 | 0.040 | Male |
| LDL cholesterol | *PER2* | 0.0034 | 0.039 | Male |
| LDL cholesterol | *PER3* | 0.0041 | 0.070 | Male |
| LDL cholesterol | *RORA* | 0.0005 | 0.053 | Male |
| LDL cholesterol | *RORC* | 0.0004 | 0.716 | Male |
| Triglycerides | *BMAL1* | 0.0001 | 0.877 | Male |
| Triglycerides | *CLOCK* | 0.0006 | 0.635 | Male |
| Triglycerides | *CRY1* | 0.0009 | 0.569 | Male |
| Triglycerides | *CRY2* | -0.0001 | 0.950 | Male |
| Triglycerides | *NR1D1* | -0.0003 | 0.825 | Male |
| Triglycerides | *NR1D2* | 0.0009 | 0.265 | Male |
| Triglycerides | *PER1* | 0.0001 | 0.175 | Male |
| Triglycerides | *PER2* | 0.0019 | 0.298 | Male |
| Triglycerides | *PER3* | 0.0046 | 0.054 | Male |
| Triglycerides | *RORA* | 0.0001 | 0.616 | Male |
| Triglycerides | *RORC* | 0.0010 | 0.419 | Male |
| BMI | *BMAL1* | -0.0002 | 0.793 | Female |
| BMI | *CLOCK* | -0.0004 | 0.642 | Female |
| BMI | *CRY1* | 0.0001 | 0.911 | Female |
| BMI | *CRY2* | -0.0004 | 0.614 | Female |
| BMI | *NR1D1* | -0.0015 | 0.122 | Female |
| BMI | *NR1D2* | -0.0005 | 0.275 | Female |
| BMI | *PER1* | -0.0001 | 0.059 | Female |
| BMI | *PER2* | 0.0000 | 0.999 | Female |
| BMI | *PER3* | -0.0008 | 0.670 | Female |
| BMI | *RORA* | -0.0001 | 0.542 | Female |
| BMI | *RORC* | -0.0006 | 0.668 | Female |
| Waist circumference | *BMAL1* | 0.0000 | 0.951 | Female |
| Waist circumference | *CLOCK* | -0.0004 | 0.659 | Female |
| Waist circumference | *CRY1* | 0.0006 | 0.615 | Female |
| Waist circumference | *CRY2* | -0.0001 | 0.855 | Female |
| Waist circumference | *NR1D1* | -0.0010 | 0.284 | Female |
| Waist circumference | *NR1D2* | -0.0004 | 0.379 | Female |
| Waist circumference | *PER1* | -0.0001 | 0.189 | Female |
| Waist circumference | *PER2* | 0.0016 | 0.239 | Female |
| Waist circumference | *PER3* | 0.0004 | 0.861 | Female |
| Waist circumference | *RORA* | -0.0001 | 0.519 | Female |
| Waist circumference | *RORC* | 0.0010 | 0.487 | Female |
| SBP | *BMAL1* | -0.0004 | 0.576 | Female |
| SBP | *CLOCK* | -0.0011 | 0.244 | Female |
| SBP | *CRY1* | -0.0010 | 0.391 | Female |
| SBP | *CRY2* | 0.0000 | 0.995 | Female |
| SBP | *NR1D1* | 0.0003 | 0.732 | Female |
| SBP | *NR1D2* | -0.0005 | 0.348 | Female |
| SBP | *PER1* | 0.0000 | 0.841 | Female |
| SBP | *PER2* | 0.0005 | 0.731 | Female |
| SBP | *PER3* | -0.0007 | 0.742 | Female |
| SBP | *RORA* | -0.0001 | 0.478 | Female |
| SBP | *RORC* | -0.0018 | 0.200 | Female |
| DBP | *BMAL1* | 0.0005 | 0.433 | Female |
| DBP | *CLOCK* | -0.0002 | 0.812 | Female |
| DBP | *CRY1* | -0.0005 | 0.698 | Female |
| DBP | *CRY2* | 0.0001 | 0.888 | Female |
| DBP | *NR1D1* | 0.0011 | 0.251 | Female |
| DBP | *NR1D2* | -0.0002 | 0.656 | Female |
| DBP | *PER1* | 0.0000 | 0.409 | Female |
| DBP | *PER2* | 0.0011 | 0.444 | Female |
| DBP | *PER3* | -0.0008 | 0.686 | Female |
| DBP | *RORA* | -0.0001 | 0.697 | Female |
| DBP | *RORC* | -0.0005 | 0.725 | Female |
| Glucose | *BMAL1* | -0.0003 | 0.657 | Female |
| Glucose | *CLOCK* | -0.0012 | 0.237 | Female |
| Glucose | *CRY1* | -0.0027 | 0.021 | Female |
| Glucose | *CRY2* | -0.0015 | 0.069 | Female |
| Glucose | *NR1D1* | 0.0000 | 0.990 | Female |
| Glucose | *NR1D2* | -0.0012 | 0.020 | Female |
| Glucose | *PER1* | -0.0001 | 0.008 | Female |
| Glucose | *PER2* | -0.0026 | 0.068 | Female |
| Glucose | *PER3* | -0.0030 | 0.142 | Female |
| Glucose | *RORA* | -0.0003 | 0.144 | Female |
| Glucose | *RORC* | -0.0007 | 0.677 | Female |
| HOMA-IR | *BMAL1* | 0.0002 | 0.772 | Female |
| HOMA-IR | *CLOCK* | 0.0008 | 0.447 | Female |
| HOMA-IR | *CRY1* | -0.0001 | 0.911 | Female |
| HOMA-IR | *CRY2* | -0.0002 | 0.822 | Female |
| HOMA-IR | *NR1D1* | 0.0008 | 0.509 | Female |
| HOMA-IR | *NR1D2* | -0.0006 | 0.297 | Female |
| HOMA-IR | *PER1* | -0.0001 | 0.109 | Female |
| HOMA-IR | *PER2* | 0.0003 | 0.853 | Female |
| HOMA-IR | *PER3* | -0.0024 | 0.276 | Female |
| HOMA-IR | *RORA* | 0.0001 | 0.791 | Female |
| HOMA-IR | *RORC* | -0.0010 | 0.554 | Female |
| Insulin | *BMAL1* | 0.0004 | 0.563 | Female |
| Insulin | *CLOCK* | 0.0011 | 0.361 | Female |
| Insulin | *CRY1* | 0.0003 | 0.834 | Female |
| Insulin | *CRY2* | -0.0007 | 0.450 | Female |
| Insulin | *NR1D1* | 0.0012 | 0.367 | Female |
| Insulin | *NR1D2* | -0.0007 | 0.225 | Female |
| Insulin | *PER1* | -0.0001 | 0.232 | Female |
| Insulin | *PER2* | 0.0003 | 0.876 | Female |
| Insulin | *PER3* | -0.0018 | 0.429 | Female |
| Insulin | *RORA* | 0.0001 | 0.611 | Female |
| Insulin | *RORC* | -0.0002 | 0.916 | Female |
| Total cholesterol | *BMAL1* | 0.0007 | 0.278 | Female |
| Total cholesterol | *CLOCK* | 0.0010 | 0.313 | Female |
| Total cholesterol | *CRY1* | 0.0010 | 0.409 | Female |
| Total cholesterol | *CRY2* | 0.0002 | 0.840 | Female |
| Total cholesterol | *NR1D1* | 0.0001 | 0.929 | Female |
| Total cholesterol | *NR1D2* | -0.0002 | 0.776 | Female |
| Total cholesterol | *PER1* | 0.0000 | 0.996 | Female |
| Total cholesterol | *PER2* | 0.0010 | 0.466 | Female |
| Total cholesterol | *PER3* | 0.0018 | 0.378 | Female |
| Total cholesterol | *RORA* | 0.0000 | 0.836 | Female |
| Total cholesterol | *RORC* | 0.0006 | 0.692 | Female |
| HDL cholesterol | *BMAL1* | 0.0001 | 0.853 | Female |
| HDL cholesterol | *CLOCK* | 0.0010 | 0.366 | Female |
| HDL cholesterol | *CRY1* | 0.0003 | 0.796 | Female |
| HDL cholesterol | *CRY2* | 0.0002 | 0.850 | Female |
| HDL cholesterol | *NR1D1* | 0.0002 | 0.856 | Female |
| HDL cholesterol | *NR1D2* | 0.0002 | 0.733 | Female |
| HDL cholesterol | *PER1* | 0.0000 | 0.983 | Female |
| HDL cholesterol | *PER2* | 0.0008 | 0.629 | Female |
| HDL cholesterol | *PER3* | 0.0024 | 0.286 | Female |
| HDL cholesterol | *RORA* | 0.0001 | 0.512 | Female |
| HDL cholesterol | *RORC* | 0.0011 | 0.542 | Female |
| LDL cholesterol | *BMAL1* | 0.0007 | 0.277 | Female |
| LDL cholesterol | *CLOCK* | 0.0008 | 0.431 | Female |
| LDL cholesterol | *CRY1* | 0.0010 | 0.377 | Female |
| LDL cholesterol | *CRY2* | -0.0006 | 0.477 | Female |
| LDL cholesterol | *NR1D1* | 0.0008 | 0.498 | Female |
| LDL cholesterol | *NR1D2* | -0.0002 | 0.717 | Female |
| LDL cholesterol | *PER1* | 0.0000 | 0.741 | Female |
| LDL cholesterol | *PER2* | 0.0007 | 0.595 | Female |
| LDL cholesterol | *PER3* | 0.0017 | 0.394 | Female |
| LDL cholesterol | *RORA* | 0.0000 | 0.888 | Female |
| LDL cholesterol | *RORC* | -0.0002 | 0.897 | Female |
| Triglycerides | *BMAL1* | 0.0003 | 0.709 | Female |
| Triglycerides | *CLOCK* | -0.0015 | 0.187 | Female |
| Triglycerides | *CRY1* | -0.0007 | 0.568 | Female |
| Triglycerides | *CRY2* | 0.0006 | 0.484 | Female |
| Triglycerides | *NR1D1* | -0.0019 | 0.128 | Female |
| Triglycerides | *NR1D2* | -0.0008 | 0.184 | Female |
| Triglycerides | *PER1* | 0.0000 | 0.857 | Female |
| Triglycerides | *PER2* | -0.0015 | 0.339 | Female |
| Triglycerides | *PER3* | -0.0048 | 0.027 | Female |
| Triglycerides | *RORA* | -0.0003 | 0.154 | Female |
| Triglycerides | *RORC* | 0.0002 | 0.921 | Female |

^1^Outcome was standardized to have a mean of 0 and standard deviation of 1. ^2^Models are adjusted for age, sex, puberty onset, smoking status, drinking status, sedentary time, moderate to vigorous physical activity time, education level of head of the household, socioeconomic status, weekday sleep duration, and InBody assessment time. Models were additionally adjusted for height when using systolic or diastolic blood pressure as the outcomes.

**
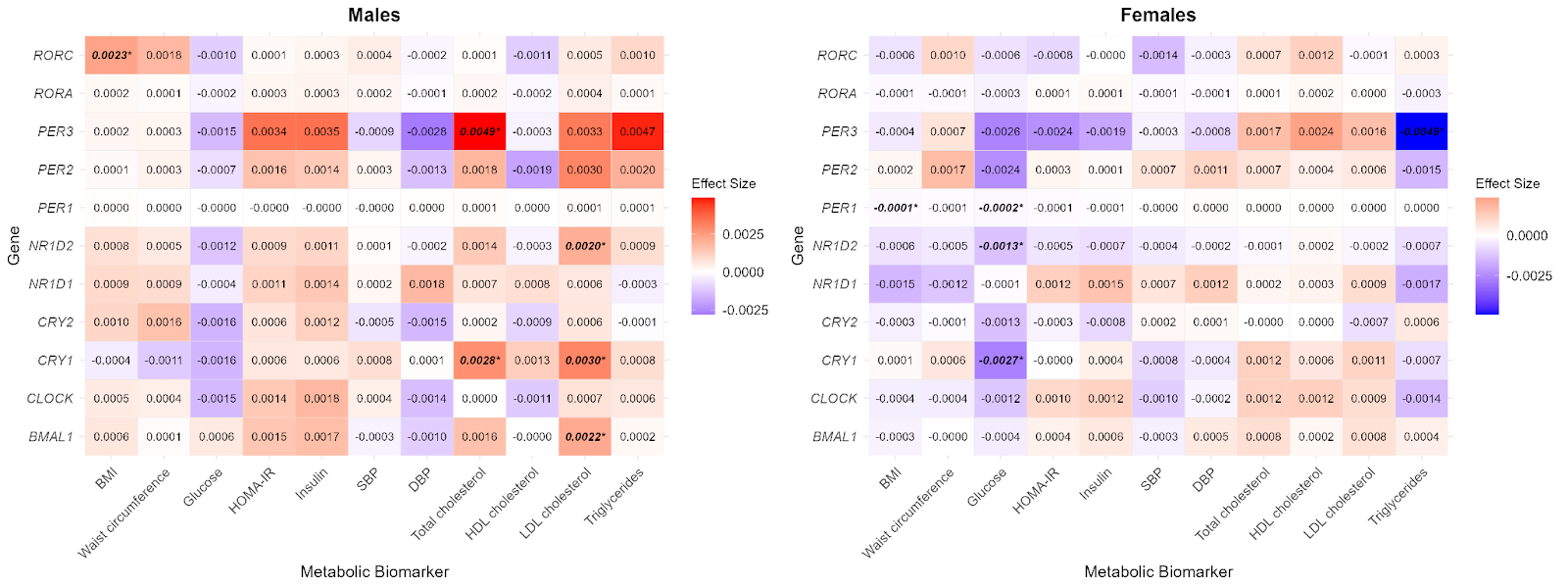
**

**Supplemental Figure 7. Associations between morning core clock gene expression and changes in metabolic biomarkers among the male (left) and females (right), additionally adjusting for dietary patterns.** Cardiometabolic risk factors were standardized to have a mean of 0 and standard deviation of 1. Models are adjusted for age, puberty onset, smoking status, drinking status, sedentary time, moderate to vigorous physical activity time, education level of head of the household, socioeconomic status, weekday sleep duration, InBody assessment time, and dietary patterns. Models were additionally adjusted for height when using systolic or diastolic blood pressure as the outcomes. Effect sizes are represented by colors and color gradients. Results shown with * are statistically significant at alpha level of 0.05.

1. BMI


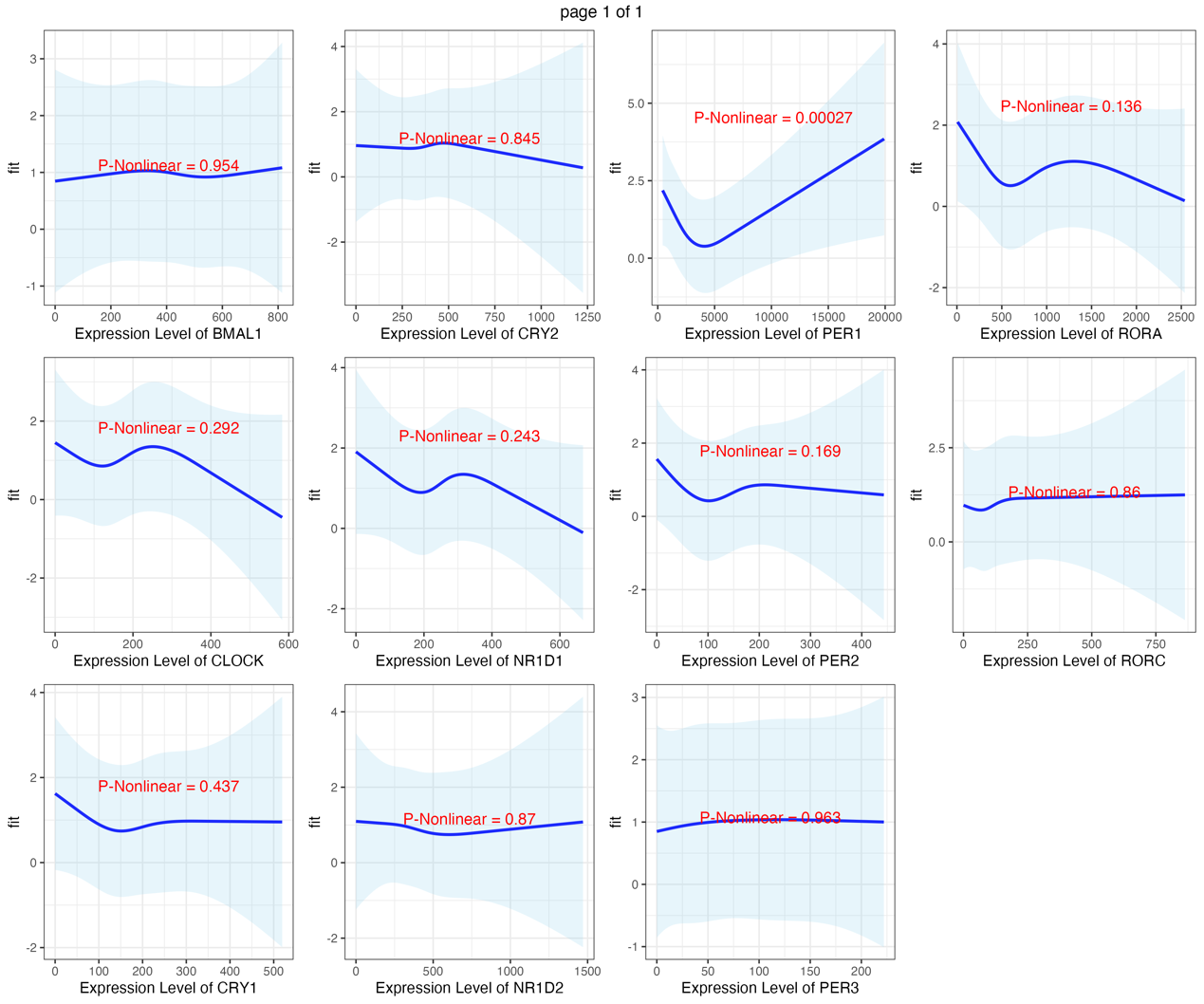


B. Waist circumference
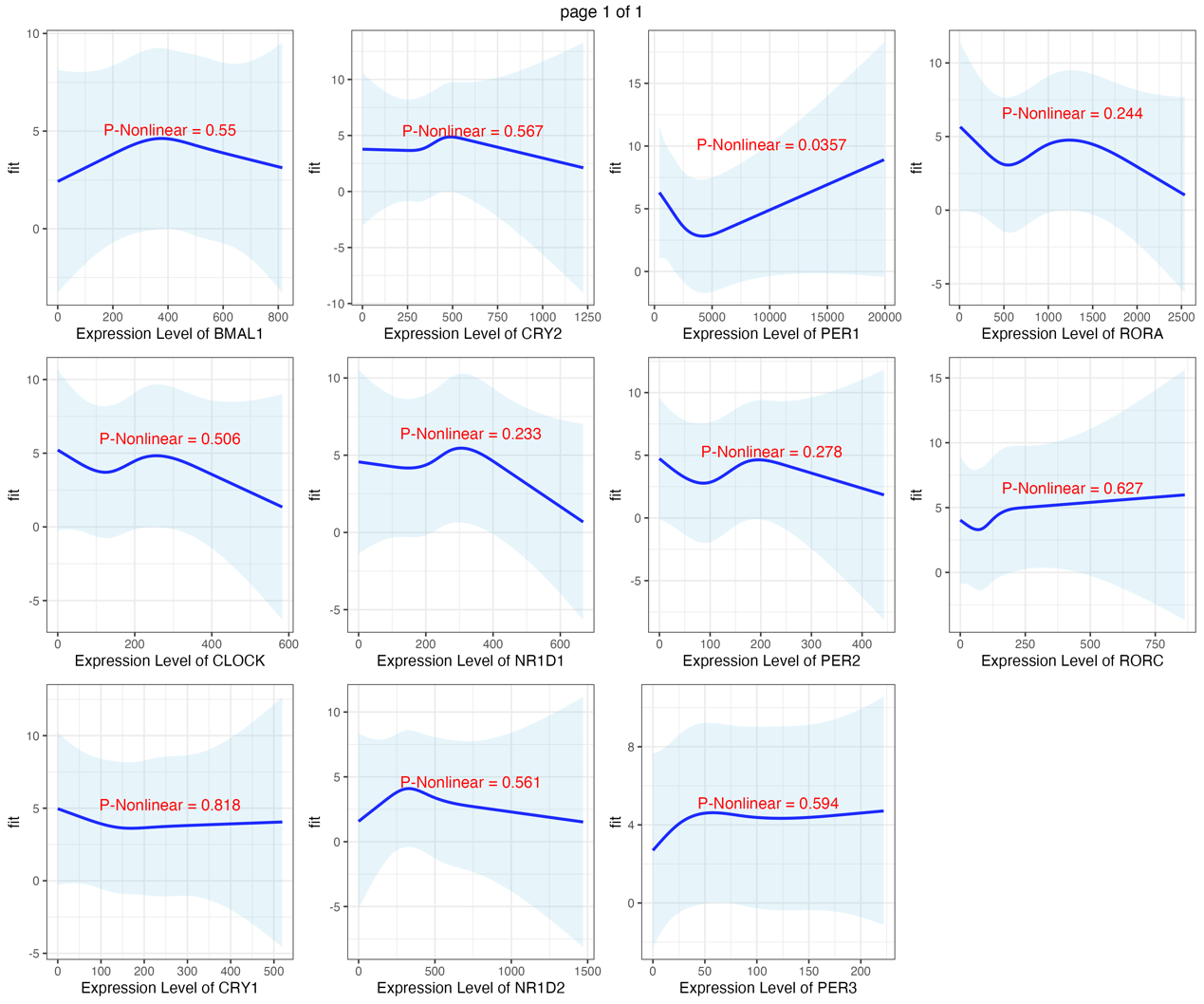


C. DBP


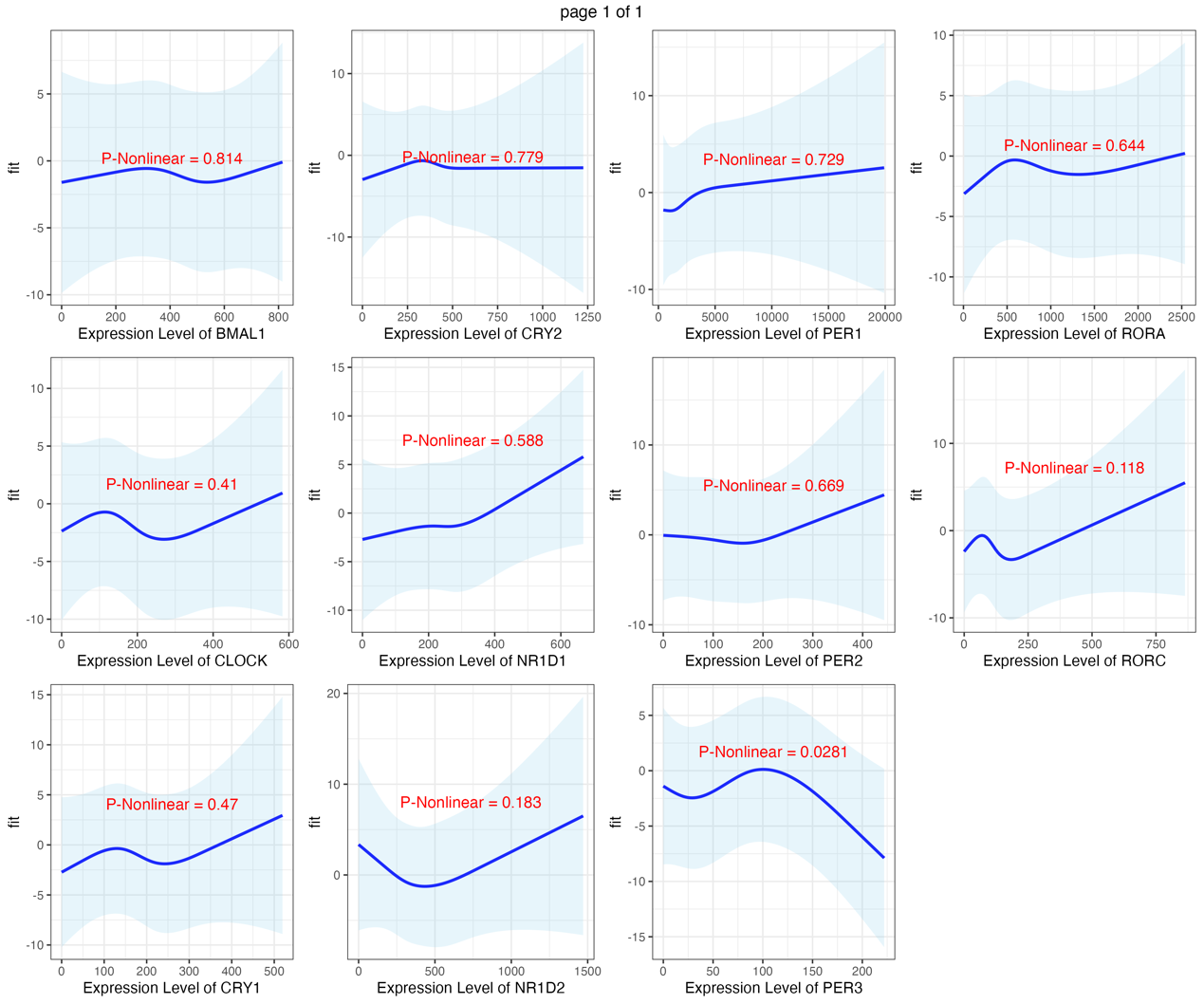


1. SBP


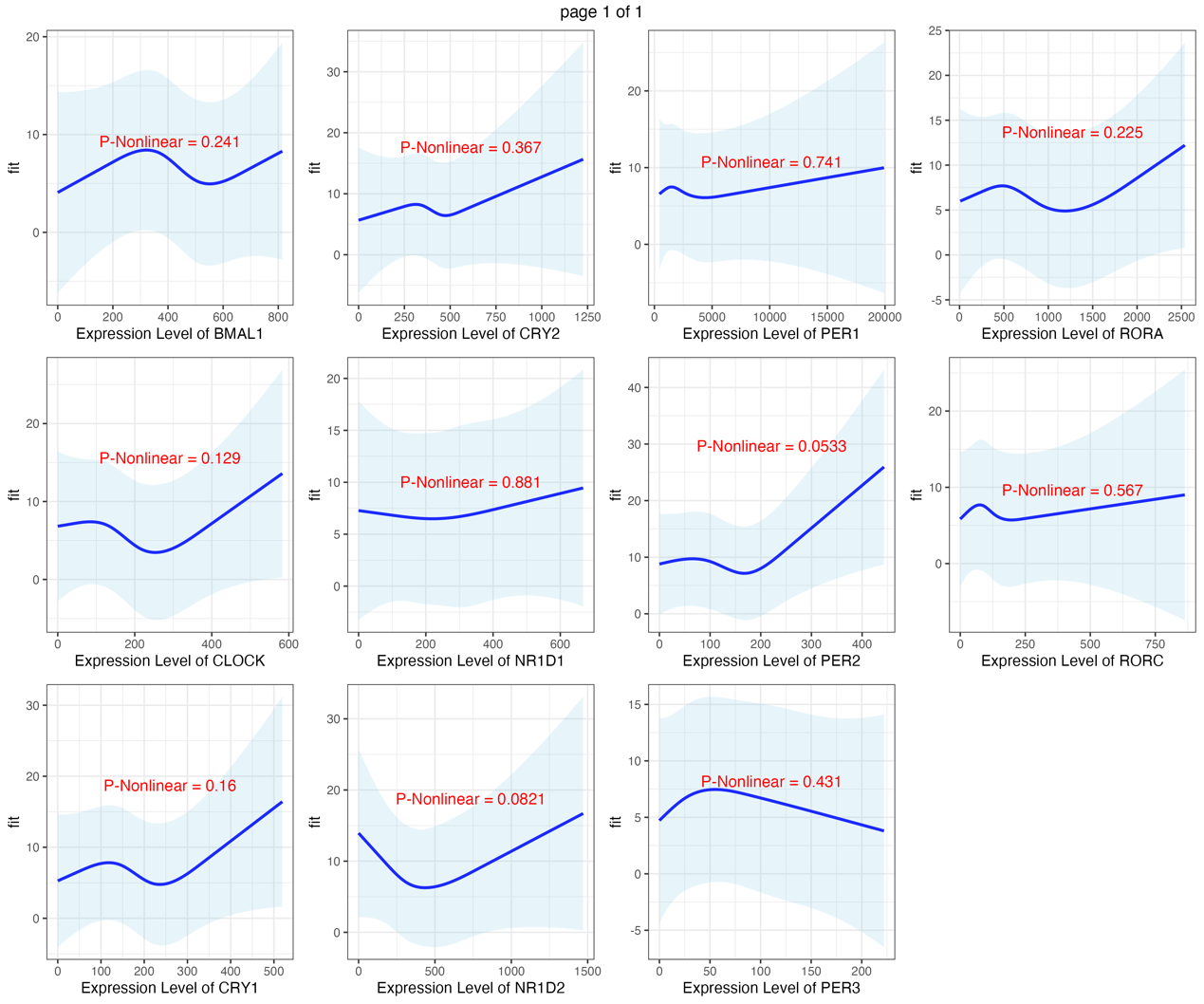


1. Glucose


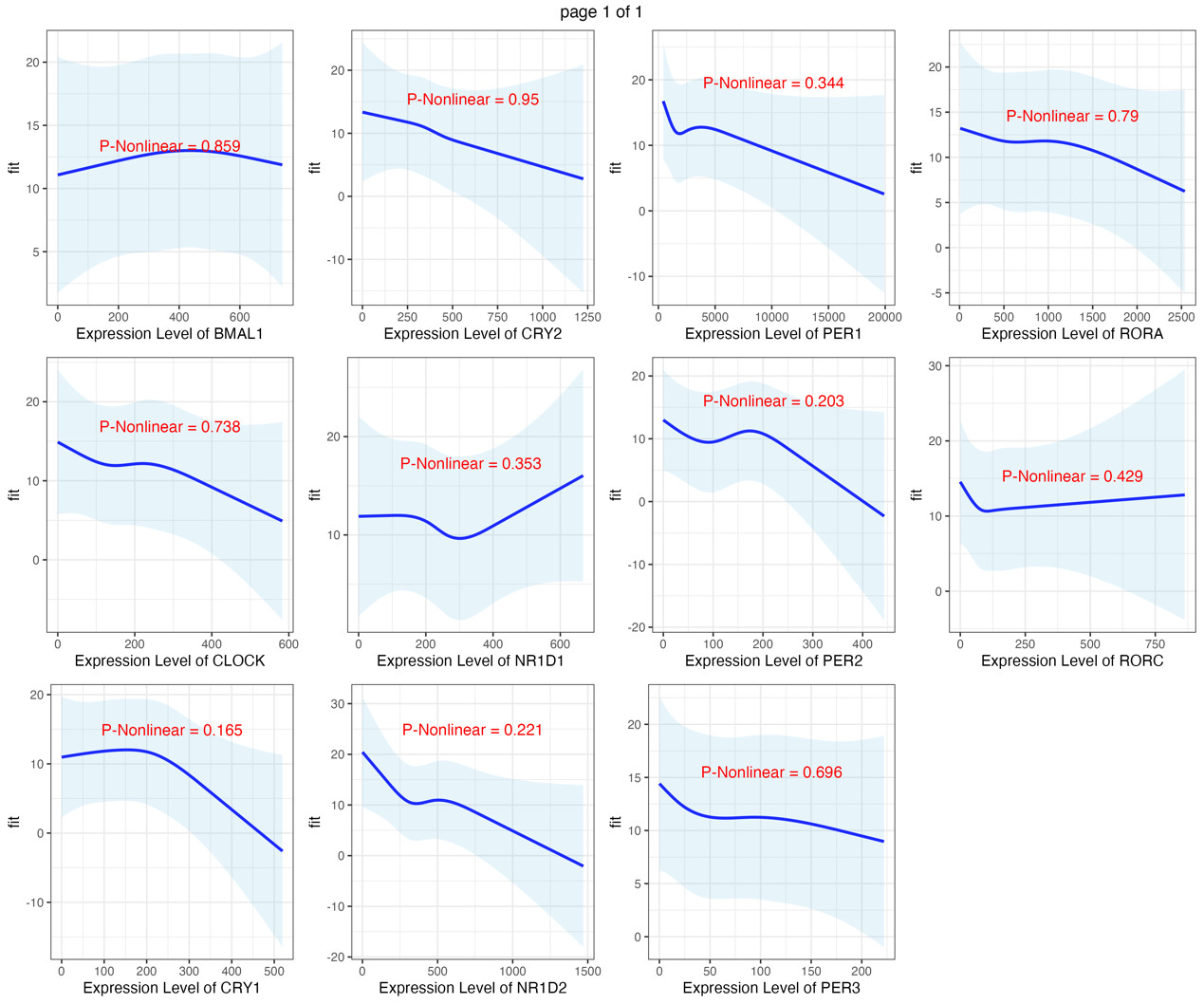


1. Insulin


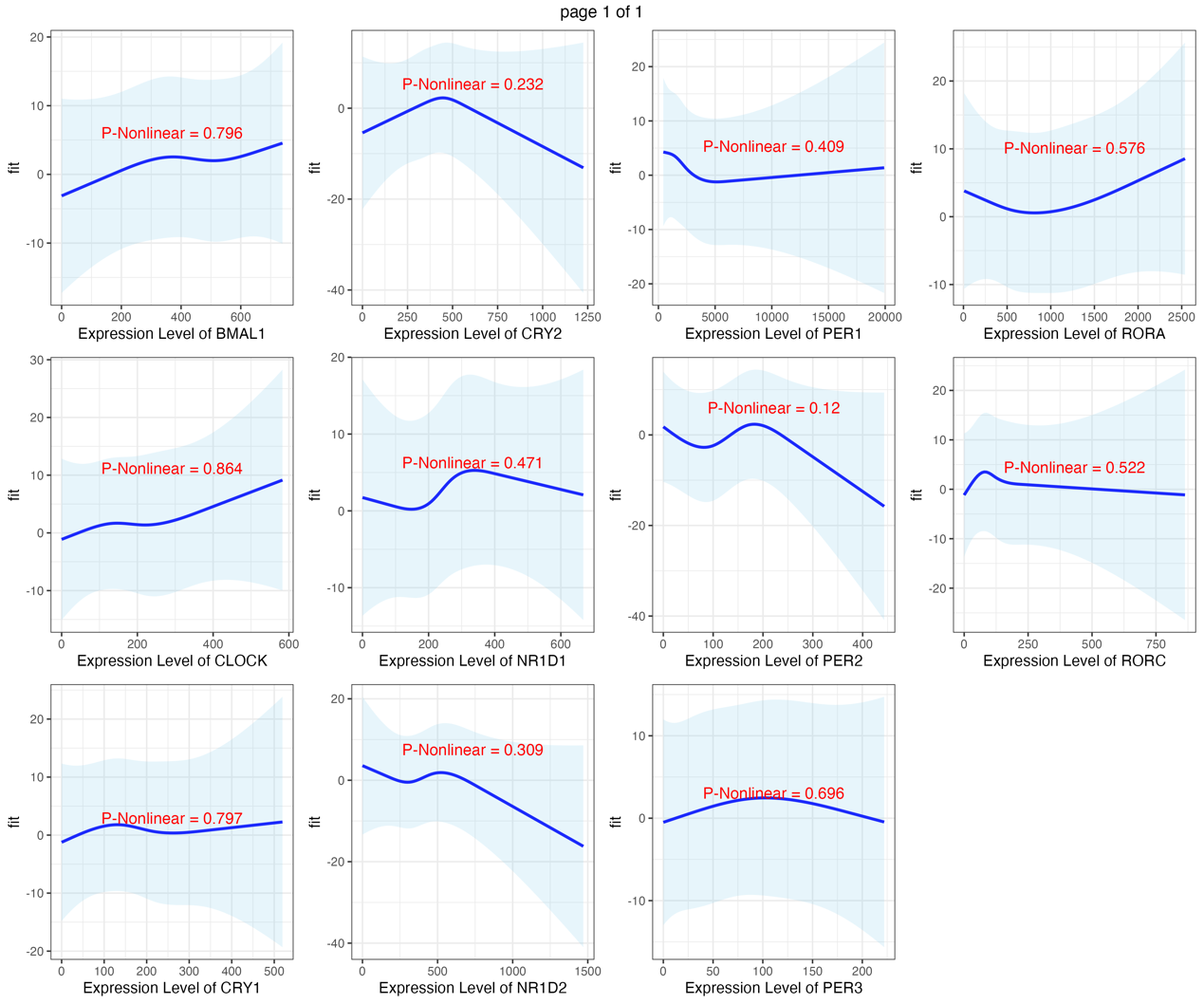


1. Homa-IR


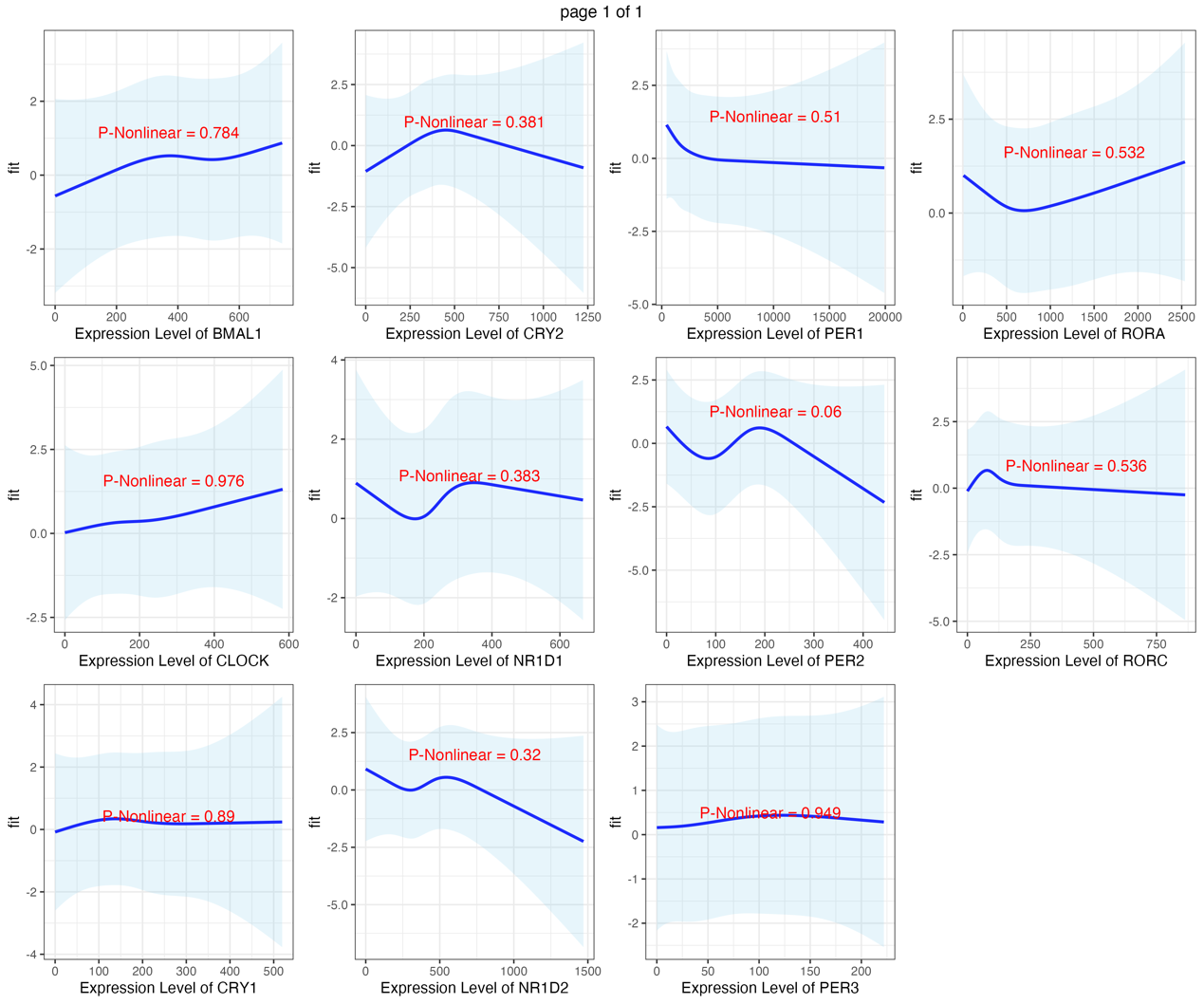


1. Total cholesterol


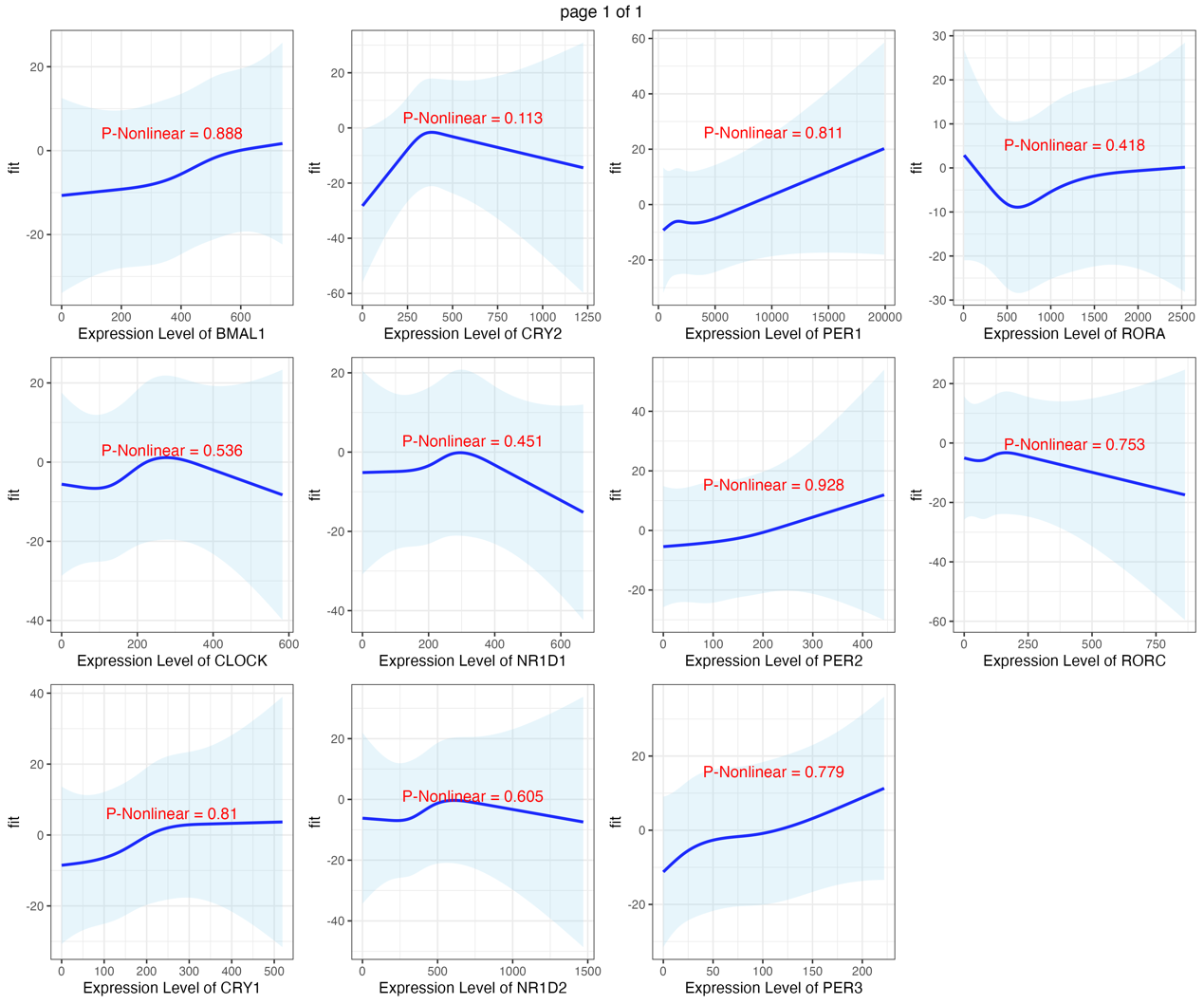


1. HDL-cholesterol


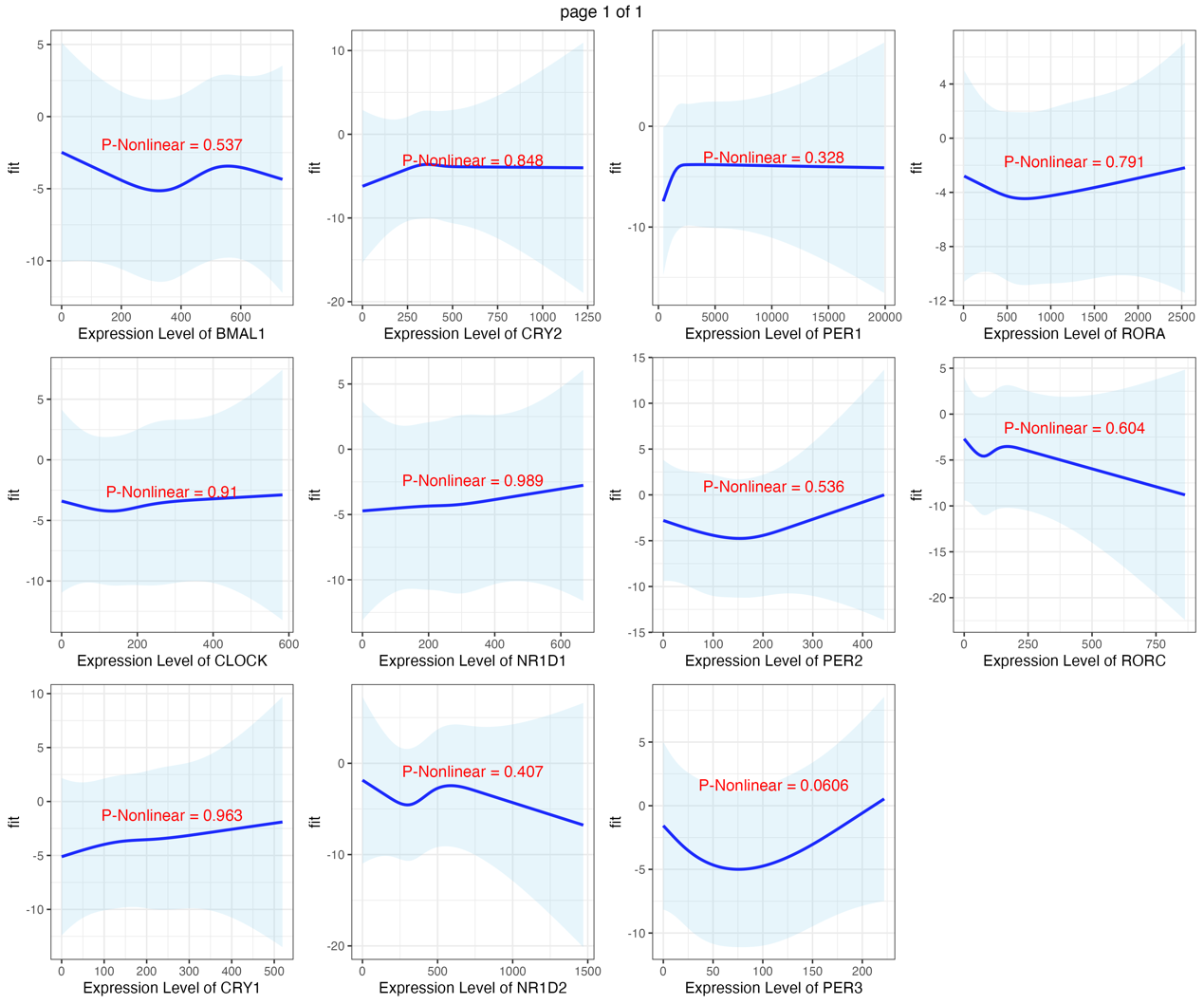


1. LDL-cholesterol


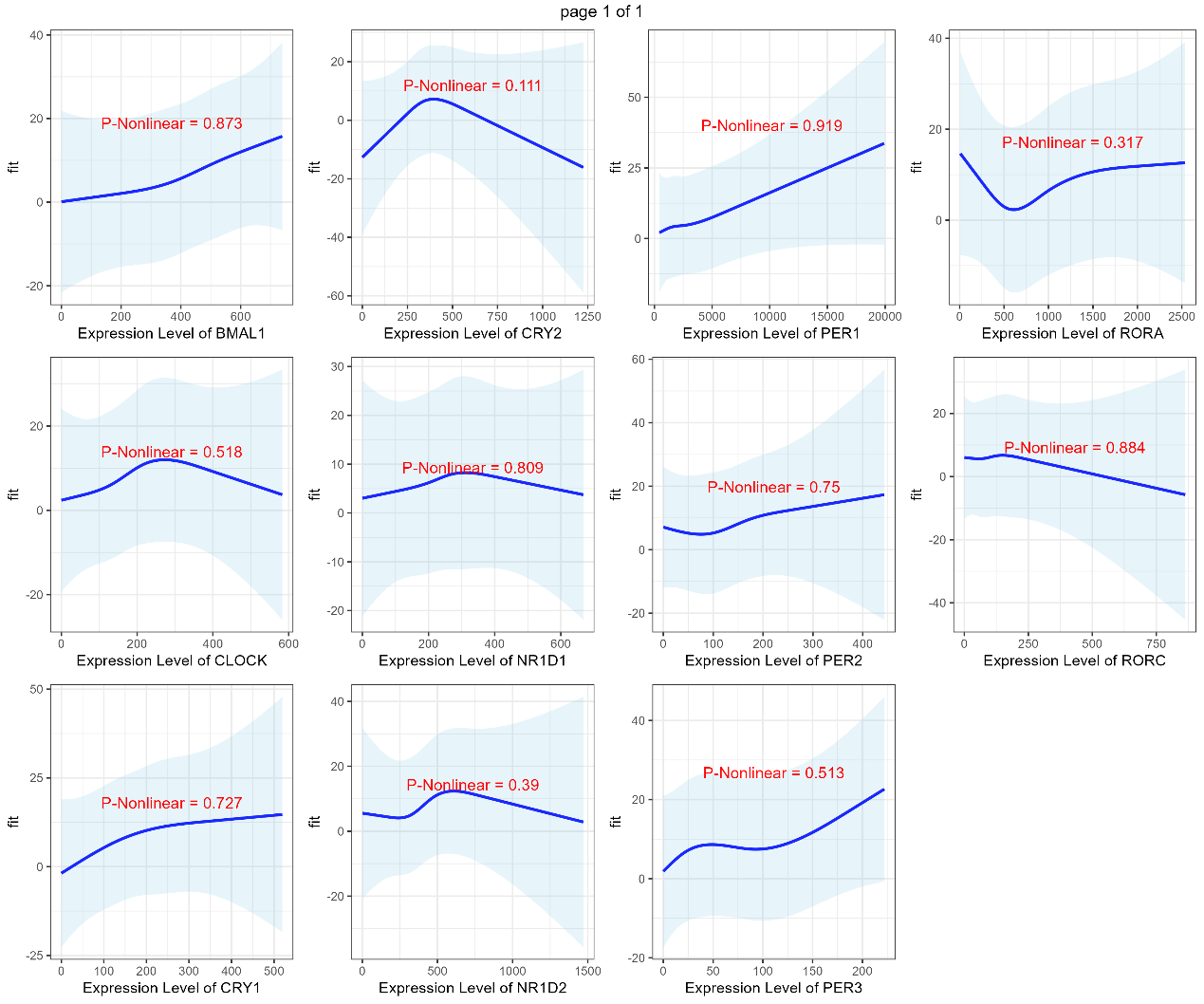


H. Triglycerides


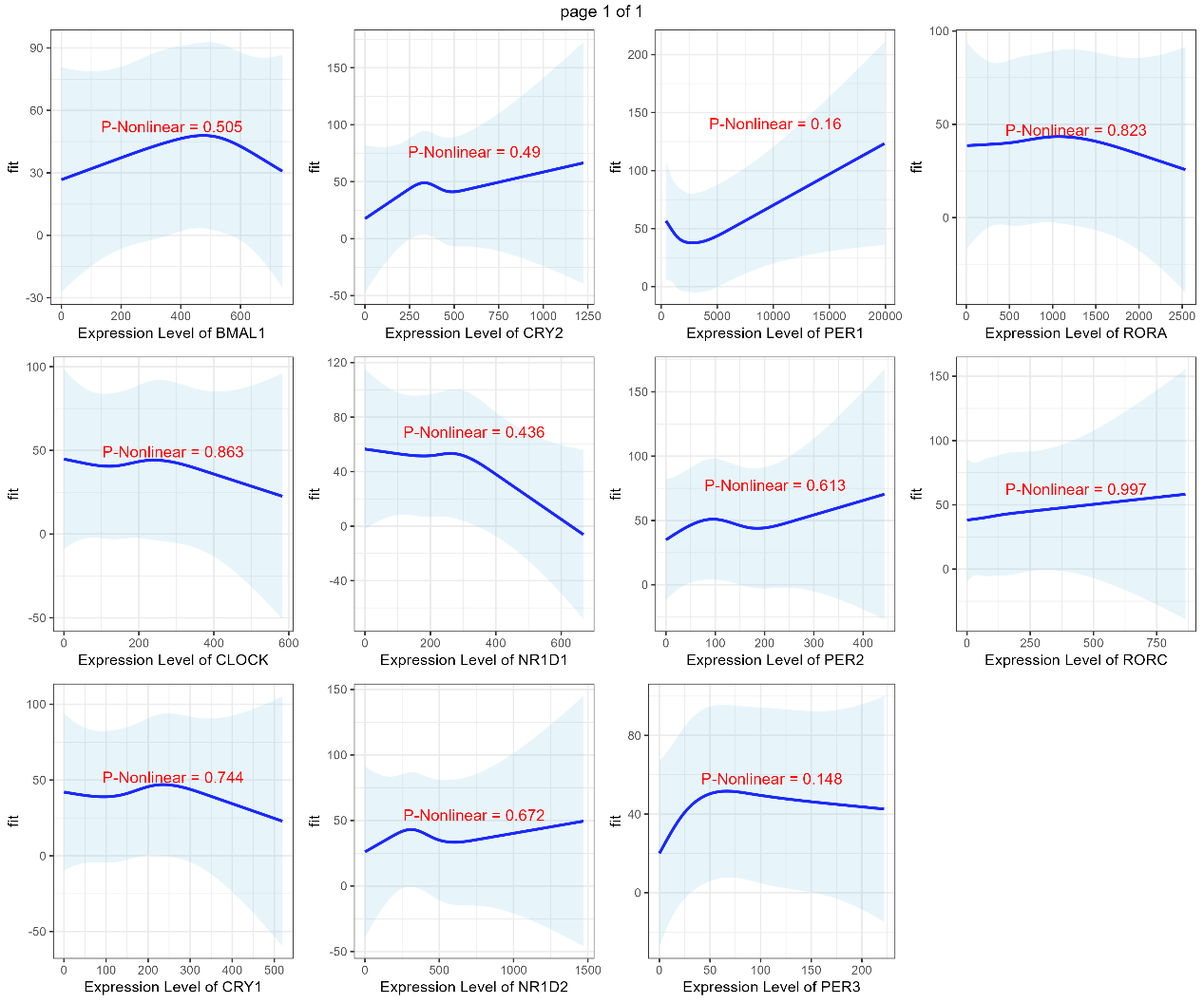


**Supplemental figure 8. Non-linear association between circadian gene expression and metabolic biomarkers.** Y axis is the changes in metabolic biomarkers from T1 to T2. Models are adjusted for age, puberty onset, smoking status, drinking status, sedentary time, moderate to vigorous physical activity time, education level of head of the household, socioeconomic status, weekday sleep duration, and InBody assessment time. Models were additionally adjusted for height when using systolic or diastolic blood pressure as the outcomes.
